# Non-specific effects of BCG and DTP vaccination on infant mortality: An analysis of birth cohorts in Ghana and Tanzania

**DOI:** 10.1016/j.vaccine.2022.04.082

**Published:** 2022-06-15

**Authors:** MK Quinn, Karen M. Edmond, Wafaie W. Fawzi, Lisa Hurt, Betty R. Kirkwood, Honorati Masanja, Alfa J. Muhihi, Sam Newton, Ramadhani A Noor, Paige L. Williams, Christopher R. Sudfeld, Emily R. Smith

**Affiliations:** a Department of Global Health and Population, Harvard T H Chan School of Public Health, Boston, MA, USA; b Department of Women and Children’s Health, Kings College London, United Kingdom; c Department of Nutrition, Harvard T H Chan School of Public Health, Boston, MA, USA; d Department of Epidemiology, Harvard T H Chan School of Public Health, Boston, MA, USA; e Division of Population Medicine, Cardiff University School of Medicine, Cardiff, UK; f Department of Epidemiology and Population Health, London School of Hygiene and Tropical Medicine, London, UK; g Ifakara Health Institute, Dar es Salaam, Tanzania; h Kwame Nkrumah University of Science and Technology, Kumasi, Ashanti, Ghana; i Kintampo Health Research Centre, Kintampo, Ghana; j Department of Biostatistics, Harvard T H Chan School of Public Health, Boston, MA, USA; k Department of Global Health, George Washington University Milken Institute School of Public Health, Washington, DC, USA; l Department of Exercise and Nutrition Sciences, George Washington University Milken Institute School of Public Health, Washington, DC, USA

**Keywords:** BCG Vaccine, DTP Vaccine, Infant mortality, Vitamin A

## Abstract

**Background:**

Vaccines may induce non-specific effects on survival and health outcomes, in addition to protection against targeted pathogens or disease. Observational evidence suggests that infant Baccillus Calmette-Guérin (BCG) vaccination may provide non-specific survival benefits, while diphtheria-tetanus-pertussis (DTP) vaccination may increase the risk of mortality. Non-specific vaccine effects have been hypothesized to modify the effect of neonatal vitamin A supplementation (NVAS) on mortality.

**Methods:**

22,955 newborns in Ghana and 31,999 newborns in Tanzania were enrolled in two parallel, randomized, double-blind, placebo-controlled trials of neonatal vitamin A supplementation from 2010 to 2014 and followed until 1-year of age. Cox proportional hazard models were used to estimate associations of BCG and DTP vaccination with infant survival.

**Results:**

BCG vaccination was associated with a decreased risk of infant mortality after controlling for confounders in both countries (Ghana adjusted hazard ratio (aHR): 0.51, 95% CI: 0.38–0.68; Tanzania aHR: 0.08, 95% CI: 0.07–0.10). Receiving a DTP vaccination was associated with a decreased risk of death (Ghana aHR: 0.39, 95% CI: 0.26–0.59; Tanzania aHR: 0.19, 95% CI: 0.16–0.22). There was no evidence of interaction between BCG or DTP vaccination status and infant sex or NVAS.

**Conclusion:**

We demonstrated that BCG and DTP vaccination were associated with decreased risk of infant mortality in Ghana and Tanzania with no evidence of interaction between DTP or BCG vaccination, NVAS, and infant sex. Our study supports global recommendations on BCG and DTP vaccination and programmatic efforts to ensure all children have access to timely vaccination.

**Clinical trials registration:** Ghana (Australian New Zealand Clinical Trials Registry (ANZCTR): ACTRN12610000582055) and Tanzania (ANZCTR: ACTRN12610000636055)

## Highlights

• BCG vaccination was associated with decreased risk of infant mortality in Tanzania and Ghana.
• DTP was also associated with decreased risk of infant mortality.
• There was no evidence of interaction between DTP or BCG vaccination, neonatal vitamin A supplementation, and infant sex on infant survival.

## 1 Introduction

Non-specific effects of vaccines are health outcomes caused by vaccination that are unrelated to the targeted pathogen or disease. There is some evidence of non-specific effects of Baccillus Calmette-Guérin (BCG) and diphtheria-tetanus-pertussis (DTP) vaccination during infancy [1]. A pooled analysis of five randomized trials in North America and Guinea Bissau demonstrated that BCG vaccination reduced the risk of child mortality by approximately 30%, which was beyond the expected mortality averted from deaths attributed to tuberculosis, and recent clinical trials have shown similar effects [1], [2], [3]. A *meta*-analysis of eight high quality cohort studies was consistent with these trial results [1]. There are no randomized trials designed to assess the effect of DTP vaccine on mortality. A *meta*-analysis of 10 observational studies in low- and middle-income countries showed substantial heterogeneity between studies; studies from Bangladesh and Papua New Guinea suggested DTP reduce mortality by 50%,[1], [4] while a study from Guinea-Bissau showed DTP increased mortality four-fold [5]. Seven of the observational studies included in the *meta*-analysis show DTP vaccination was linked with an increased risk of all-cause mortality [1].

Infant sex has been hypothesized to modify the magnitude of non-specific effects of vaccines. There is not consistent evidence that effect of BCG was modified by sex in multiple trials [1], [2], [3], [6], [7], [8], but the issue remains an open question in the case of DTP [1]. Only one of twelve studies that have examined the sex-specific effects of DTP on all-cause mortality have found that female infants fare worse than males [9]. While eight additional observational studies show a similar trend, three others show the opposite[1]. The *meta*-analysis conducted at the request of the World Health Organization (WHO) following a recommendation of the Strategic Advisory Group of Experts (SAGE) concluded that existing data are inconclusive regarding potential sex-specific differences in the effect of DTP on all-cause mortality [1].

It has also been suggested that these non-specific effects could be amplified by vitamin A supplementation [10]. The World Health Organization (WHO) currently recommends vitamin A supplementation for children 6–59 months of age living in communities where vitamin A deficiency is a public health problem [11]; however, there is no such recommendation for infants younger than 6 months. Randomized trials of vitamin A supplementation in neonates have not shown overall survival benefits; however, recent *meta*-analyses illustrate there is heterogeneity across populations and suggest some newborns may benefit from supplementation [12], [13], [14]. One hypothesis to explain the heterogeneity of trial results is that the effect of neonatal vitamin A may differ by vaccination status due to a sex-specific interaction with heterologous vaccine effects [15]. To date there has been only one randomized trial designed specifically to analyze the interaction between BCG vaccination and vitamin A. This trial was conducted in Guinea-Bissau and found that BCG vaccination did not modify the effect of vitamin A on survival [16]. The evidence regarding the interaction between vitamin A and DTP vaccination on child mortality is less clear, since there have been no randomized trials investigating this interaction. One observational analysis of a vitamin A supplementation trial in Guinea-Bissau found that vitamin A supplementation was associated with increased mortality (hazard ratio (HR): 2.19, 95% CI: 1.09–4.38) only within the subgroup of female infants who had received DTP [17]. However, a similar study in Tamil Nadu found no interaction [18].

Differences in underlying vitamin A deficiency (VAD) may also explain heterogeneity in the results. Trials conducted in contexts with higher prevalence of VAD tend to show positive effects of vitamin A supplementation on mortality, while trials in contexts with low prevalence of VAD have found no effect [14]. This is further supported by data from Tanzania showing that maternal vitamin A supplementation and maternal dietary intake of vitamin A modified the effect of neonatal vitamin A supplementation on mortality [19]. However, maternal vitamin A supplementation did not modify the effect in trials in Bangladesh or Zimbabwe [20], [21]. Overall, factors that may contribute to potential differential effects of neonatal vitamin A on mortality remains unclear.

The overall aim of this analysis was to examine the non-specific effects of BCG and DTP vaccination on infant mortality and the potential interaction by infant sex and neonatal vitamin A supplementation based on data from two large, randomized controlled trials of neonatal vitamin A supplementation conducted in Ghana and Tanzania.

## 2 Methods

### 2.1 Study design and participants

This prospective cohort study used data from two parallel, randomized double-blind, placebo- controlled trials of neonatal vitamin A supplementation conducted in Ghana (Australian New Zealand Clinical Trials Registry (ANZCTR): ACTRN12610000582055) and Tanzania (ANZCTR: ACTRN12610000636055) [22], [23]. The assessment of whether the effect of vitamin A on mortality was modified by DTP was included a priori in the original trial protocol [24]. The analysis of the association BCG and DTP with mortality was a posthoc analysis.

The trials were conducted from 2010 to 2014 and were implemented with a similar protocol [24]. Briefly, infants were eligible for the trials if they were able to feed orally and families planned to live in the study area for 6 months. In Ghana, liveborn neonates were recruited in 7 contiguous districts in central, rural Ghana. In Tanzania, pregnant mothers were recruited from antenatal clinics, labor wards, or population-based pregnancy surveillance in the Dar es Salaam and Morogoro regions [22], [23]. All participants provided written consent for participation.

### 2.2 Randomization and data collection

In Ghana and Tanzania, eligible infants were randomized at birth to either placebo or single-dose oral capsules of 50,000 IU of vitamin A. Over the course of one year, infants were visited at home by field interviewers, and data on vaccination dates were collected from immunization records and if immunization records were unavailable, vaccination status was collected through caregiver interview. Research staff verified the information for all infant deaths and performed a verbal autopsy. In Ghana, follow up visits occurred monthly until 12 months of age. In Tanzania, follow up visits occurred at 1, 3, 6, and 12 months. All study protocols have been described in detail elsewhere [24].

The study protocols were approved by the Ghana Health Service Ethical Review Committee, the ethics committees of the Kintampo Health Research Centre, Ghana, the London School of Hygiene and Tropical Medicine, UK, the Institutional Review Boards of the Harvard T.H. Chan School of Public Health, Ifakara Health Institute, Medical Research Coordinating Council of Tanzania, and by the WHO Ethical Review Committee.

### 2.3 Statistical analysis

We first examined the potential non-specific effects of BCG and DTP vaccination by assessing the association between vaccination and survival using time-varying Cox proportional hazard models. The underlying time scale was days of age. Schoenfeld residuals were plotted to assess the proportional hazards assumption. Participants contributed person-time to unvaccinated and vaccinated periods, and vaccination status was updated over time, at the time the vaccine was received. This has been referred to as retrospective updating and was chosen to minimize exposure misclassification and selection bias. This method in comparison to other methods used for this type of data are described further in supplemental figures S1-S3 and tables S1-S2.

Because participants were only eligible for the DTP vaccine after 30 days of life, our analysis of DTP vaccination included only infants who had survived to 30 days. Additionally, the time origin was set at 30 days because analyzing DTP from birth introduces potential immortal time bias since deaths occurring before eligibility would be attributed to being unvaccinated.

The adjusted models included the birthweight and sociodemographic confounders collected at birth. For the Ghana data, we modeled continuous birthweight with cubic splines with knots at 1.5, 2, 2.5, and 3.5 kg. We also included: head of household (mother, father, grandmother, grandfather, other), household religion (Christian, Muslim, None, Other), maternal age (<20, 20–24, 25–29, 30–34, 35–39, ≥40), maternal education (None, Primary, Secondary, Post-secondary), multiple or singleton birth, number of living children in household (0, 1, 2, 3 + ), number of children in household who have died (0, 1, 2, 3 + ), place of birth (Compound, Facility, Other), site ID (1–4), wealth quintile, delivery type (vaginal or caesarean), and maternal megadose of vitamin A. For the Tanzania models, we included the same variables, but sites were Ifakara and Dar es Salaam and educational categories were limited to none, primary, or secondary and higher. Missing covariate values were imputed in the Ghana and Tanzania datasets. The primary analysis was restricted to those with complete information on whether they received BCG for the BCG analysis or whether they received their first DTP vaccination for the DTP analysis. Vaccine information was considered missing unless there was either a yes or no on the vaccination card or confirmation by caregiver interview. Analyses for BCG and DTP were conducted separately, and data was only excluded if the relevant vaccine was missing information. Sensitivity analyses to test the effect of different assumptions regarding missing data are described below.

We next examined the potential interaction between neonatal vitamin A supplementation (NVAS) and BCG or DTP vaccination and examined the three -way interaction between vitamin A supplementation, vaccination, and infant sex. To do this, we fit time-varying cox proportional hazard models for the effect of vitamin A status on survival allowing for interaction by vaccination status. Similar to the analysis of vaccination alone, vaccine status was allowed to vary with time and models were adjusted for the same baseline covariates to control for confounding between vaccination and survival. Interaction p-values were calculated using likelihood ratio tests comparing the model with the interaction of vitamin A and vaccination to the model without the interaction term. We also conducted stratified analyses by sex to evaluate whether there were sex-specific interactions between NVAS and vaccination status. We tested whether the three-way interaction p-value was statistically significant using a likelihood ratio test comparing a model with the three-way interaction for vitamin A or placebo, vaccination status, and sex to a model with only two-way interactions between vitamin A or placebo, vaccination status, and sex. Finally, we examined whether there was effect modification of the association between DTP and survival by a previous BCG vaccination.

All analyses were conducted in R 3.5.1, and p-values<0.05 were considered statistically significant.

### 2.4 Sensitivity analyses

We conducted sensitivity analyses to investigate how robust the results were to missing vaccination data, particularly for infants who died. We investigated three scenarios for the missing vaccination data that represented the extreme range of possibilities: 1, assuming all participants with missing vaccination status or date of vaccination were vaccinated on day 0 for BCG or day 30 for DTP (contributing to only vaccinated person-time); 2, assuming all participants missing vaccination status or vaccination date data were vaccinated at the mode of 1 day for BCG in Ghana and Tanzania and 45 days and 31 days for DTP in Ghana and Tanzania, respectively (contributing to both unvaccinated and vaccinated person-time); 3, assuming none of the participants who were missing vaccination data were ever vaccinated during follow-up (contributing to only unvaccinated person-time) (Supplemental Table S3).

## 3 Results

The Ghana trial enrolled 22,955 newborns, and the Tanzania trial enrolled 31,999 newborns. Supplemental Tables S4 and S5 presents baseline characteristics of the study cohorts stratified by randomized vitamin A and placebo regimen; both cohorts were balanced in terms of baseline characteristics across randomized arms. There were 699 deaths in Ghana (30 per 1,000 live births) and 1,112 deaths in Tanzania (35 per 1,000 live births). Of included participants with known vaccination status, 98% received BCG and 99% received the first DTP dose in Ghana; 97 % received BCG and 92% received the first DTP dose in Tanzania. BCG vaccination data was missing for 604 (2.6%) in Ghana and 1,700 (5.3%) in Tanzania. DTP vaccination data was missing for 223 (1.0%) in Ghana and 96 (0.3%) in Tanzania. The construction of the dataset is shown in supplemental figures S4 and S5. When population characteristics were compared across vaccination status, those who were never vaccinated with BCG tended to be born to mothers with less education, outside of facilities, and their mothers were less likely to receive a megadose of vitamin A in Ghana (Supplemental Table S6). Infants never vaccinated with DTP in Ghana tended to have lower birthweights and to be born to mothers in lower wealth quintiles and with less education (Supplemental Table S7). In Tanzania, those who were never vaccinated with BCG tended to be born to mothers in lower wealth quintiles (Supplemental Table S8), and infants who were vaccinated late with DTP were more likely to be born to younger mothers and mothers in lower wealth quintiles (Supplemental Table S9).

We first examined the association of BCG vaccination and the risk of mortality overall and by infant sex (Table 1). In Cox proportional hazard models allowing for time-varying vaccination status and adjusting for covariates, BCG vaccination was associated with a 49% reduced risk of death in Ghana (HR: 0.51, 95% CI: 0.38, 0.68) and a 92% reduced risk of death in Tanzania (HR: 0.08, 95% CI: 0.07, 0.10). There was no evidence that the association of BCG vaccination with mortality differed by infant sex (Ghana: p = 0.70, Tanzania: p = 0.32) or NVAS (Ghana: p = 0.44, Tanzania: p = 0.17). We present sensitivity analysis considering unknown vaccination status in Supplemental Table S10. If we assumed all those with unknown vaccination status did not receive BCG vaccination, the direction of the association did not change in the results for Tanzania. However, we found that in Ghana, BCG was associated with increased mortality if all unknown vaccinations were assumed to occur at baseline (HR: 4.58, 95% CI: 3.53, 5.95) or 1 day (HR: 2.71, 95% CI: 2.10, 3.48) (Supplemental Table S10).

**Table 1:** Association of BCG vaccination with infant mortality (from 0 to 365 days), overall and stratified by infant sex.

		BCG Vaccinated		Not BCG Vaccinated		**Unadjusted HR (95% CI) for Mortality**^a^	**Adjusted HR (95% CI) for Mortality**^b^
		Number of deaths	Number of infant months at risk	Number of deaths	Number of infant months at risk		
**Ghana**							
Full population		382	249,104	96	16,643	0.38 (0.29, 0.51)	0.51 (0.38, 0.68)
Population by sex	Female Infants	189	123,070	44	8,059	0.40 (0.27, 0.59)	0.52 (0.35, 0.79)
	Male Infants	193	126,034	52	8,584	0.37 (0.25, 0.55)	0.48 (0.32, 0.73)
**Tanzania**							
Full population		573	341,332	203	11,189	0.08 (0.07, 0.10)	0.08 (0.07, 0.10)
Population by sex	Female Infants	257	162,595	83	5,348	0.09 (0.07, 0.13)	0.10 (0.07, 0.13)
	Male Infants	316	178,701	120	5,841	0.08 (0.06, 0.10)	0.08 (0.06, 0.10)

a All analyses were conducted using Cox proportional hazard models allowing for time-varying vaccination status.

b Adjusted models controlled for continuous birthweight (with spline knots at 1.5, 2, 2.5, and 3.5 kg), head of household (mother, father, grandmother, grandfather, other), household religion (Christian, Muslim, None, Traditional African), maternal age (<20, 20–24, 25–29, 30–34, 35–39, ≥40), maternal education (None, Primary, Secondary for Tanzania, and additionally Post-secondary for Ghana), multiple or singleton birth, number of living children in household (0, 1, 2, 3 + ), number of children in household who have died (0, 1, 2, 3 + ), place of birth (Home, Facility, Other), site ID (1–4 for Ghana and 1–2 for Tanzania), wealth quintile, delivery type (vaginal or caesarean), and maternal megadose of vitamin A.

The association of the first DTP vaccination with infant mortality among infants surviving to 30 days is presented in Table 2. DTP vaccination was associated with a 61% reduced risk of death in Ghana (HR: 0.39, 95% CI: 0.26, 0.59) and an 81% reduced risk of death in Tanzania (HR: 0.19, 95% CI: 0.16, 0.22). Again, there was no evidence that the association of DTP vaccination with mortality differed by infant sex (Ghana: p = 0.71, Tanzania: p = 0.29) or NVAS (Ghana: p = 0.18, Tanzania: p = 0.80). To account for unknown DTP vaccination status, we calculated bounds on the measures of association we observed for extreme cases (Supplemental Table S11). The results from Tanzania remained consistent even after accounting for missing vaccine status or timing. However, we found that in Ghana, DTP was associated with increased mortality if all missing vaccinations were assumed to occur at the 30-day mark (HR: 1.56, 95% CI: 0.99, 2.47). However, the measures of association for Ghana remained essentially unchanged if we assumed missing vaccination for Ghana happened at the mode (45 days) (HR: 0.59, 95% CI: 0.39, 0.89) (Supplemental Table S11).

**Table 2:** Association of DTP vaccination with infant mortality (from 30 to 365 days), overall and stratified by infant sex.

		DTP Vaccinated		Not DTP Vaccinated		**Unadjusted HR (95% CI) for Mortality**^a^	**Adjusted HR (95% CI) for Mortality**^b^
		Number of deaths	Number of infant months at risk	Number of deaths	Number of infant months at risk		
**Ghana**							
Full population		298	219,008	69	24,860	0.34 (0.23, 0.52)	0.39 (0.26, 0.59)
Population by sex	Female Infants	149	108,115	32	12,122	0.51 (0.27, 0.95)	0.59 (0.32, 1.10)
	Male Infants	149	110,894	37	12,738	0.25 (0.15, 0.42)	0.28 (0.16, 0.47)
**Tanzania**							
Full population		402	282,709	234	34,066	0.19 (0.16, 0.22)	0.19 (0.16, 0.22)
Population by sex	Female Infants	186	134,448	99	16,270	0.21 (0.16, 0.27)	0.22 (0.16, 0.28)
	Male Infants	216	148,229	135	17,783	0.17 (0.14, 0.22)	0.17 (0.13, 0.21)

a All analyses were conducted using Cox proportional hazard models allowing for time-varying vaccination status.

b Adjusted models controlled for continuous birthweight (with spline knots at 1.5, 2, 2.5, and 3.5 kg), head of household (mother, father, grandmother, grandfather, other), household religion (Christian, Muslim, None, Traditional African), maternal age (<20, 20–24, 25–29, 30–34, 35–39, ≥40), maternal education (None, Primary, Secondary for Tanzania, and additionally Post-secondary for Ghana), multiple or singleton birth, number of living children in household (0, 1, 2, 3 + ), number of children in household who have died (0, 1, 2, 3 + ), place of birth (Home, Facility, Other), site ID (1–4 for Ghana and 1–2 for Tanzania), wealth quintile, delivery type (vaginal or caesarean), and maternal megadose of vitamin A.

We examined the potential three-way interaction between BCG vaccination, NVAS and by infant sex (Table 3). In both Ghana and Tanzania, there was no evidence of interactions between randomized vitamin A supplementation and BCG vaccination (p-values: 0.45 and 0.17, respectively). For Ghana and Tanzania combined, we found no effect of vitamin A supplementation on mortality for both BCG unvaccinated time and BCG vaccinated time (Supplemental Table S12, Supplemental Figure S6a and S6b). Furthermore, there was no evidence of interaction between vitamin A and BCG vaccination when either population was stratified by sex (Table 3). There was no evidence of a three-way interaction between vitamin A supplementation, DTP vaccination, and sex (p = 0.49 for Ghana and p = 0.75 for Tanzania).

**Table 3:** The adjusted effect of Vitamin A Supplementation on Infant Mortality, stratified by BCG Vaccination Status and Infant Sex.

		**Vitamin A**		**Placebo**		**HR (95% CI) for Mortality**^a^	**P-value for BCG-Vitamin A Interaction**^b^
		Number of deaths	Number of infant months	Number of deaths	Number of infant months		
**Ghana**							
**Overall Population**	Unvaccinated Time (BCG)	53	8,264	43	8,378	1.26 (0.84–1.89)	0.45
	Vaccinated Time (BCG)	195	12,4193	187	124,911	1.06 (0.86–1.29)	
**Female Infants**	Unvaccinated Time (BCG)	25	3,963	19	4,096	1.28 (0.70–2.34)	0.35
	Vaccinated Time (BCG)	90	60,993	99	62,077	0.93 (0.70–1.24)	
**Male Infants**	Unvaccinated Time (BCG)	28	4,302	24	4,283	1.22 (0.71–2.15)	0.94
	Vaccinated Time (BCG)	105	63,200	88	62,834	1.20 (0.96–1.60)	
**Tanzania**							
**Overall Population**	Unvaccinated Time (BCG)	115	5,661	88	5,528	1.31 (0.99–1.73)	0.17
	Vaccinated Time (BCG)	291	170,382	282	170,950	1.04 (0.88–1.23)	
**Female Infants**	Unvaccinated Time (BCG)	51	2,714	32	2,634	1.51 (0.97–2.36)	0.30
	Vaccinated Time (BCG)	136	80,636	121	81,959	1.16 (0.91–1.48)	
**Male Infants**	Unvaccinated Time (BCG)	64	2,947	56	2,894	1.16 (0.81–1.66)	0.37
	Vaccinated Time (BCG)	155	89,722	161	88,979	0.95 (0.76–1.19)	

a All hazard ratios are calculated using Cox proportional hazards models allowing for time-varying vaccination status and controlling for continuous birthweight (with spline knots at 1.5, 2, 2.5, and 3.5 kg), head of household (mother, father, grandmother, grandfather, other), household religion (Christian, Muslim, None, Traditional African), maternal age (<20, 20–24, 25–29, 30–34, 35–39, ≥40), maternal education (None, Primary, Secondary for Tanzania, and additionally Post-secondary for Ghana), multiple or singleton birth, number of living children in household (0, 1, 2, 3 + ), number of children in household who have died (0, 1, 2, 3 + ), place of birth (Home, Facility, Other), site ID (1–4 for Ghana and 1–2 for Tanzania), wealth quintile, delivery type (vaginal or caesarean), and maternal megadose of vitamin A.

b Three-way interaction between Vitamin A status, BCG vaccination, and sex was not significant (Ghana: p = 0.49, Tanzania: p = 0.75).

**Fig. 1:**
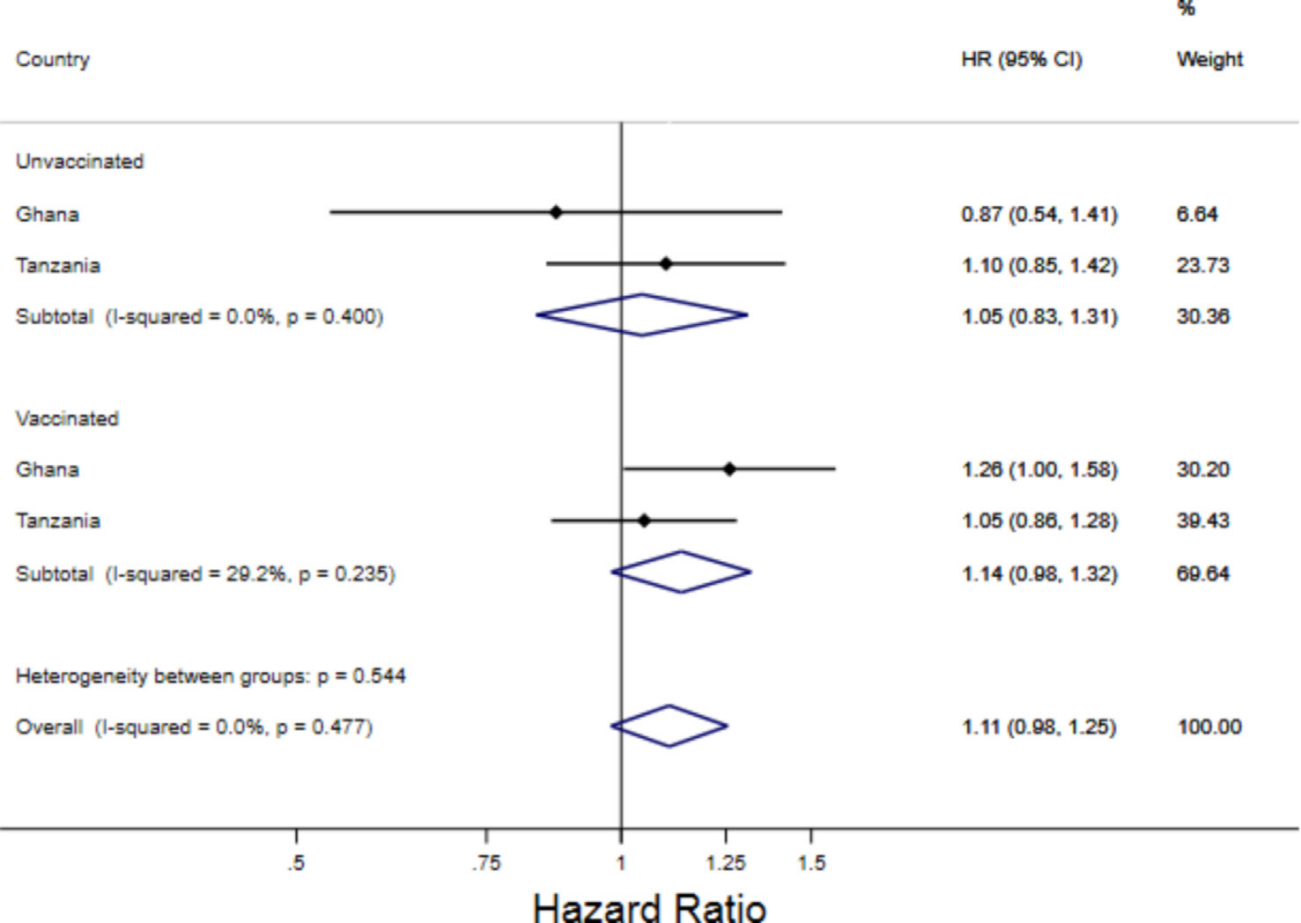
**Combined forest plot of the adjusted effect of vitamin A supplementation on infant mortality, stratified by DTP vaccination status**Fig. 1**Description:** These estimates correspond with estimates presented in Table 3 of the main paper. Hazard Ratios were combined by through a fixed-effect metanalysis of the adjusted estimates from Tanzania and Ghana. All hazard ratios are calculated using Cox proportional hazards models allowing for time-varying vaccination status and are controlled for continuous birthweight (with spline knots at 1.5, 2, 2.5, and 3.5 kg), head of household (mother, father, grandmother, grandfather, other), household religion (Christian, Muslim, None, Traditional African), maternal age (<20, 20–24, 25–29, 30–34, 35–39, ≥40), maternal education (None, Primary, Secondary for Tanzania, and additionally Post-secondary for Ghana), multiple or singleton birth, number of living children in household (0, 1, 2, 3 + ), number of children in household who have died (0, 1, 2, 3 + ), place of birth (Home, Facility, Other), site ID (1–4 for Ghana and 1–2 for Tanzania), wealth quintile, delivery type (vaginal or caesarean), and maternal megadose of vitamin A. See supplemental table S13.

**Fig. 2:**
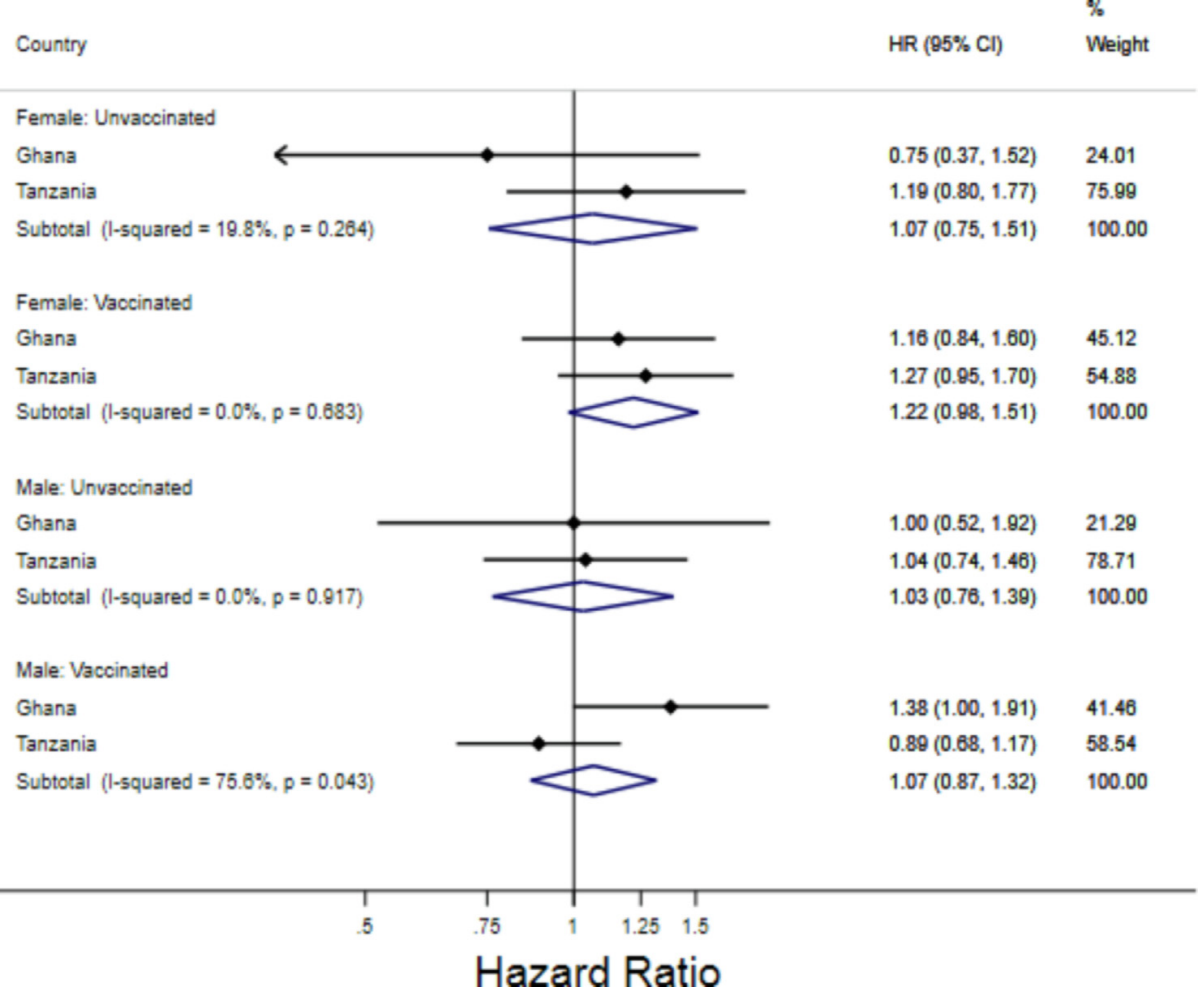
**Combined forest plot of the adjusted effect of vitamin A supplementation on infant mortality, stratified by DTP vaccination status and sex**Fig. 2**Description:** These estimates correspond with estimates presented in Table 3 of the main paper. Hazard Ratios were combined by through a fixed-effect metanalysis of the adjusted estimates from Tanzania and Ghana. All hazard ratios are calculated using Cox proportional hazards models allowing for time-varying vaccination status and are controlled for continuous birthweight (with spline knots at 1.5, 2, 2.5, and 3.5 kg), head of household (mother, father, grandmother, grandfather, other), household religion (Christian, Muslim, None, Traditional African), maternal age (<20, 20–24, 25–29, 30–34, 35–39, ≥40), maternal education (None, Primary, Secondary for Tanzania, and additionally Post-secondary for Ghana), multiple or singleton birth, number of living children in household (0, 1, 2, 3 + ), number of children in household who have died (0, 1, 2, 3 + ), place of birth (Home, Facility, Other), site ID (1–4 for Ghana and 1–2 for Tanzania), wealth quintile, delivery type (vaginal or caesarean), and maternal megadose of vitamin A. See supplemental table S13.

**Fig. 3:**
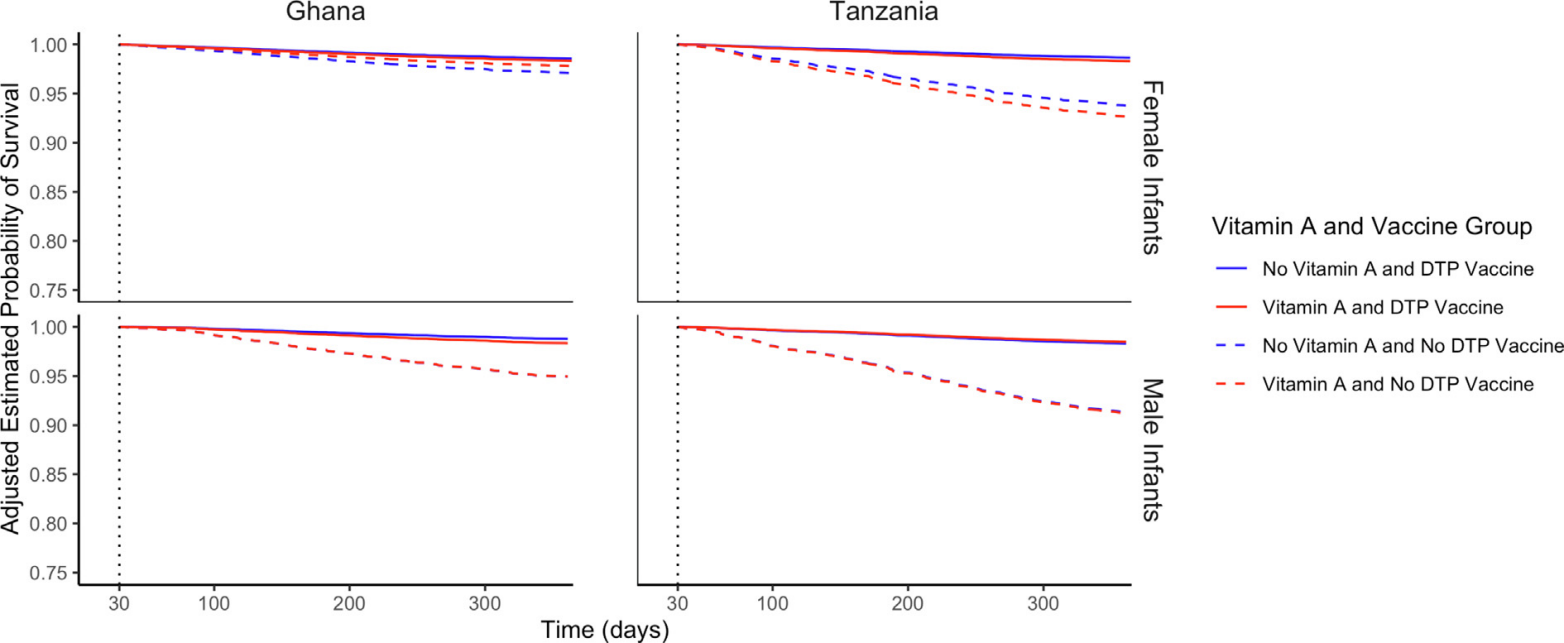
**Adjusted Survival Curves among Female and Male Infants by Vitamin A and DTP Vaccination Beginning at 30 Days**Fig. 3**Description:** Cox proportional hazards models were used to predict survival curves, accounting for covariates using weighted averages to get an overall estimate for each vitamin A supplementation and vaccination subgroup. Covariates included continuous birthweight (with spline knots at 1.5, 2, 2.5, and 3.5 kg), head of household (mother, father, grandmother, grandfather, other), household religion (Christian, Muslim, None, Traditional African), maternal age (<20, 20–24, 25–29, 30–34, 35–39, ≥40), maternal education (None, Primary, Secondary for Tanzania, and additionally Post-secondary for Ghana), multiple or singleton birth, number of living children in household (0, 1, 2, 3 + ), number of children in household who have died (0, 1, 2, 3 + ), place of birth (Home, Facility, Other), site ID (1–4 for Ghana and 1–2 for Tanzania), wealth quintile, delivery type (vaginal or caesarean), and maternal megadose of vitamin A.

We then examined the potential interaction of NVAS with DTP vaccination and infant sex (Table 4). For Ghana and Tanzania combined, we found no effect of vitamin A supplementation on mortality for both DTP unvaccinated time and DTP vaccinated time (Fig. 1, Fig. 2, Supplemental Table S13). We found no evidence of interactions between vitamin A supplementation and periods of vaccination (Ghana: p = 0.18, Tanzania: p = 0.80). This was true for both female infants (Ghana: p = 0.26, Tanzania: p = 0.78) and male infants (Ghana: p = 0.38, Tanzania: p = 0.50). We additionally found no evidence of a three-way interaction between vitamin A supplementation, DTP vaccination, and sex (p = 0.84 for Ghana and p = 0.53 for Tanzania). Based on adjusted survival curves among female and male infants by vitamin A and DTP vaccination beginning at 30 days for Ghana and Tanzania (Fig. 3), it is evident that receiving a DTP vaccination was consistently associated with improved survival regardless of vitamin A supplementation status and regardless of infant sex.

**Table 4:** The adjusted effect of Vitamin A Supplementation on Infant Mortality, stratified by DTP Vaccination and Infant Sex, from 30 to 365 days.

		**Vitamin A**		**Placebo**		**HR (95% CI) for Mortality**^a^	**P-value for DTP-Vitamin A Interaction**^b^
		Number of deaths	Number of infant months	Number of deaths	Number of infant months		
**Ghana**							
**Overall Population**	Unvaccinated Time (DTP)	31	12,327	38	12,533	0.87 (0.54–1.41)	0.18
	Vaccinated Time (DTP)	165	109,301	133	109,707	1.26 (1.00–1.58)	
**Female Infants**	Unvaccinated Time (DTP)	13	5,898	19	6,224	0.75 (0.37–1.52)	0.26
	Vaccinated Time (DTP)	79	53,589	70	54,526	1.16 (0.84–1.60)	
**Male Infants**	Unvaccinated Time (DTP)	18	6,430	19	6,309	1.00 (0.52–1.92)	0.38
	Vaccinated Time (DTP)	86	55,712	63	55,181	1.38 (0.99–1.91)	
**Tanzania**							
**Overall Population**	Unvaccinated Time (DTP)	123	17,100	111	16,966	1.10 (0.85–1.42)	0.80
	Vaccinated Time (DTP)	205	141,196	197	141,513	1.05 (0.87–1.28)	
**Female Infants**	Unvaccinated Time (DTP)	54	8,157	45	8,113	1.19 (0.80–1.77)	0.78
	Vaccinated Time (DTP)	103	66,783	83	67,665	1.27 (0.95–1.70)	
**Male Infants**	Unvaccinated Time (DTP)	69	8,943	66	8,840	1.04 (0.74–1.46)	0.50
	Vaccinated Time (DTP)	102	74,391	114	73,838	0.89 (0.68–1.17)	

a All hazard ratios are calculated using Cox proportional hazards models allowing for time-varying vaccination status and controlling for continuous birthweight (with spline knots at 1.5, 2, 2.5, and 3.5 kg), head of household (mother, father, grandmother, grandfather, other), household religion (Christian, Muslim, None, Traditional African), maternal age (<20, 20–24, 25–29, 30–34, 35–39, ≥40), maternal education (None, Primary, Secondary for Tanzania, and additionally Post-secondary for Ghana), multiple or singleton birth, number of living children in household (0, 1, 2, 3 + ), number of children in household who have died (0, 1, 2, 3 + ), place of birth (Home, Facility, Other), site ID (1–4 for Ghana and 1–2 for Tanzania), wealth quintile, delivery type (vaginal or caesarean), and maternal megadose of vitamin A.

b Three-way interaction between Vitamin A status, DTP vaccination, and sex was not significant (Ghana: p = 0.83, Tanzania: p = 0.53).

To again account for unknown BCG or DTP vaccination status, we calculated bounds for the interaction analysis (Supplemental Tables S14-S19). There were no changes in the direction of the associations compared to our complete-case analysis (Table 4) when all unknown vaccines were assumed to be given at the earliest recommended date, assumed to be given at the mode, or assumed to not be given.

There was evidence of effect modification of the association of DTP vaccination and survival by a previous BCG vaccine, but the effect of DTP in both BCG groups was protective. The stratified effects are shown in Supplemental Table S20.

## 4 Discussion

Our findings suggest that BCG and DTP vaccination were associated with a lower risk of infant mortality, and the magnitude of the associations were consistent with beneficial non-specific effects of vaccination. There was no evidence that these non-specific vaccine effects for either BCG or DTP were modified by NVAS or infant sex and these findings were consistent in both Ghana and Tanzania. Our study is the largest cohort to investigate the nonspecific effects of DTP and the potential interaction with vitamin A on infant mortality and provides results from two different contexts within sub-Saharan Africa. Frequent follow up visits, monthly in Ghana and quarterly in Tanzania, minimized potential measurement error in the exposure and outcome assessment. Furthermore, sensitivity analyses demonstrated that our findings were robust to limitations from unknown vaccination data for the association between DTP and mortality and for vaccines modifying the effect of NVAS on mortality were robust to limitations from missing vaccination data. Further, a major strength was that NVAS was randomized.

Our finding that BCG is associated with 29–92% reduction in mortality is consistent with current epidemiologic literature. The SAGE *meta*-analysis of five clinical trials comparing BCG to placebo or to delayed vaccination estimated that BCG vaccination resulted in a 30% (95% CI: −1% to 51%) lower risk of mortality [1]. Recent clinical trials have found similar effects [2], [3], though different strains of BCG vaccine likely have different non-specific effects [6], and protective effects were not observed in some populations [7], [8]. In addition, a *meta*-analysis of 13 observational studies of BCG found that the vaccine was associated with a 53% (95% CI: −1% to 68%) lower risk of mortality. Our observational study found similarly lower risk of mortality in Ghana (49% lower, 95% CI: 32% to 62%), and much lower risk of mortality in Tanzania (92% lower, 95% CI: 90% to 93%). The low hazard ratios that we observed in Tanzania may be related to differential misclassification of vaccine status, which might be expected to be more severe with the longer time between study visits as compared to Ghana and other similar studies. Our study did not collect data on the strain of BCG administered, and thus cannot compare our results directly to studies of the same strain. Our analysis did not find a differential in the association of BCG vaccination and mortality by sex. This is consistent with the epidemiological literature on sex-specific BCG vaccination. The SAGE *meta*-analysis of sex-specific BCG nonspecific effects found nine studies with a ratio of relative risks 1.02 for males to females (95% CI: 0.73 to 1.41) [1]. Some recent trials have found that BCG tended to be more protective for male infant outcomes [2], [3], [6]. Others found BCG tended to be more protective for females [7], [8], but no recent trials found significant effect modification of BCG on mortality by sex.

We found that DTP was associated with a 61%-81% lowered risk of mortality. No previous clinical trial data are available to determine whether DTP impacts all-cause mortality, but the SAGE *meta*-analysis of 10 observational studies (deemed at high risk of bias) found a non-significant tendency towards increased risk of death associated with the DTP vaccination (RR: 1.38, 95% CI: 0.92, 2.08) [1]. Among the epidemiologic studies on DTP, there is a divide between those with a finding an increased risk of mortality,[5], [25], [26], [27], [28] and those finding a lower risk or no significant risk of mortality, consistent with our results.[4], [9], [18], [29], [30] This study investigated only the first DTP vaccination. Future studies may help to understand the contributions of the second and third vaccination to all-cause mortality. Our study is the largest to date and indicates DTP is linked to improved survival and therefore supports continued expansion of DTP coverage. We observed different estimates of the association between DTP and survival and BCG and survival in Ghana and Tanzania. This could be due to different contributions of immortal time bias, misclassification, or unmeasured confounding in Ghana and Tanzania. It could also be due to non-specific effects of vaccines presenting differently in populations with different childhood disease prevalences. However, we consistently saw positive associations between BCG vaccination and survival and DTP vaccination and survival in both countries.

Furthermore, our analysis did not find differential effects of DTP vaccination by sex. Although observational studies have shown mixed results, the SAGE analysis reported that there was no sex differential for the effect of DTP on mortality (ratio of relative risks for males to females: 0.72, 95% CI: 0.46 – 1.14) [1]. The pooled ratio of relative risks is challenging to interpret because it reflects both studies that suggest both sexes have beneficial non-specific effects of vaccination, but the magnitude of benefit was greater in males compared to females, and there are studies that found increased risk of mortality in females and decreased risk of mortality in males. There are three studies in India, Senegal, and Guinea Bissau that have shown a tendency towards excess mortality among female infants [31], [32], [33]. Generally, studies that use the landmark method find stronger sex-differentials than studies using retrospective updating [34]. Our study finds DTP was associated with reduced risk of mortality for both male and female infants in Ghana and Tanzania and there was no statistically significant interaction by sex; however, data from both countries show a trend similar to the *meta*-analysis whereby benefits of DTP may be greater for male infants.

Evidence on the potential interaction between neonatal vitamin A and nonspecific effects of vaccines on mortality is limited to five studies, and the results are mixed [16], [17], [18], [35], [36]. A reanalysis of a NVAS clinical trial among normal weight infants (>2500 g) in Guinea-Bissau suggested that there may be an interaction between sex and NVAS among infants who received a DTP vaccination [17]. This study found that NVAS was associated with increased risk of mortality among the subgroup of female infants vaccinated with DTP, whereas this was not found among male infants. However, the study did not find statistically significant interaction and age was a proxy for vaccination status. This makes it difficult to isolate whether vaccination status, age, or another factor associated with age in this cohort were driving the association with survival. It has also been suggested that analyses such as these suffer from selection bias due to left and right censoring [37]. A trial focused on low birth weight infants (<2500 g) in Guinea-Bissau found a non-significant tendency for vitamin A to be more harmful for females receiving DTP when the data was stratified, but no evidence of statistical interaction between vitamin A, sex, and vaccination [16]. A later study assessing the effects of high and low-dose NVAS in Guinea-Bissau similarly did not find statistically significant interactions, although it was likely to have been underpowered [35]. Furthermore, an analysis retesting the hypothesis in Guinea-Bissau did not find evidence that early DTP in female infants was associated with increased mortality [36]. Thus, the majority of existing evidence for an interaction between vitamin A supplementation, DTP, and sex is limited to non-significant trends in the data and may be observed due to chance. The study most similar to our analysis, an observational cohort of a randomized controlled trial of NVAS conducted in Tamil Nadu, did not find a significant interaction between DTP, vitamin A, and sex on mortality [18]. Our analysis was better powered than previous studies to investigate the interaction of vitamin A and vaccination on neonatal survival and allowed for modeling time-varying vaccination to ensure accurate exposure definition [17], [35], [38].

Our study may be limited by biases consistent with common sources of bias in the literature on non-specific vaccine effects and vitamin A: differential misclassification of exposures, confounding, and selection bias [39]. Differential misclassification of vaccination may be the methodological driver of studies finding that DTP vaccination is protective of mortality. This arises because vaccination status is more often unknown for children who died. In our study, BCG vaccination status was unknown for 3% and 5% of all participants, and DTP vaccination status was unknown for 1% and<1% of analyzed participants in Ghana and Tanzania, respectively. It is possible that more differential misclassification occurred in the Tanzania dataset due to less frequent data collection visits.

Two analytic methods that have been used in the literature are “landmark analyses” and “retrospective analysis”, and the choice of analysis has been shown to change the direction of the association between the investigated vaccine and mortality [40], [41], [42]. In the retrospective analyses used in our study, vaccine exposure status changes at the date of vaccination. This is considered “retrospective” since data on vaccination is collected after the vaccination occurs. In the landmark approach, the vaccination exposure status changes only at study visits when the vaccination card is available. However, this approach introduces additional nondifferential misclassification since time between the date of vaccination and study visit will be incorrectly classified as unvaccinated. For this reason, we chose to use the actual recorded date of vaccination and then compute upper and lower uncertainty bounds to address missing exposure data for deaths, and we argue this approach is most appropriate to address the potential bias [43], [44].

Furthermore, observational studies of non-specific effects of vaccination are at high-risk of confounding, particularly due to infant health [45]. While our study controlled for birthweight which can act as a partial proxy for infant health due to the strong association between birthweight and infant mortality,[46] we could not completely control for infant health status. Also, we controlled for many socioeconomic factors, however, we cannot rule out residual or unmeasured confounding which would result in stronger protective associations. Another potential confounder that we did not systematically assess was maternal HIV status. It is possible that vaccination timing is different for infants born to mothers living with HIV, and infants would likely have higher risk mortality [47]. As a result, unmeasured confounding by maternal HIV status is possible and may affect the Tanzania trial estimates to a greater extent than Ghana trial due to a higher prevalence of HIV.

Selection bias also likely plays a role in the results seen in many studies of non-specific effects of vaccines. Selection bias can be induced by setting the origin of the survival model at later time points in infancy, [37] since those who are unlikely to be vaccinated are also likely to be those at a higher risk of death due to underlying health status. Our DTP results are only generalizable to the population surviving to 30 days for incident DTP vaccination. However, vaccine schedules do not recommend vaccination prior to 30 days of life and by considering only this population, we were able to minimize potential selection biasing effects of including outcomes of infants who were not yet eligible for vaccination. Nevertheless, it is important to note that while we carefully considered the potential biases associated with our analytic decisions, the observational nature of our study limits our ability to make causal determinations on the non-specific effects of vaccination.

## 5 Conclusions

Overall, our study offers rigorous evidence that BCG and DTP vaccination were associated with a decreased risk of infant mortality, and the magnitude of the associations were consistent with beneficial non-specific effects of vaccination. There was no evidence of interaction between DTP or BCG vaccination, NVAS, and infant sex. Our study supports global recommendations on BCG and DTP vaccination and programmatic efforts to ensure all children indicated for vaccination have access to timely vaccination.

Funding.

The parent studies were funded by the Bill & Melinda Gates Foundation. MK Quinn was supported by the National Institutes of Health (NIH) under Award Number T32AI007358. The funders had no role in study design, data collection, data interpretation, writing of the report, or the decision to submit the article for publication.

## Declaration of Competing Interest

The authors declare that they have no known competing financial interests or personal relationships that could have appeared to influence the work reported in this paper.
