# Supplementary material for: Non-specific effects of BCG and DTP vaccination on infant mortality: An analysis of birth cohorts in Ghana and Tanzania
Source: Vaccine. 2022 Jun 15;40(27):3737–45. doi: 10.1016/j.vaccine.2022.04.082 (PMC9194613; doi:10.1016/j.vaccine.2022.04.082)
Supplement: Supplementary data 1 [file mmc1.docx]

Supplementary Data

[Methods 3](#_Toc100919971)

[**Method Selection and Bias** 3](#_Toc100919972)

[**Table S1: Description of three proposed methods** 4](#_Toc100919973)

[**Figure S1: Immortal time bias and alignment of eligibility, treatment, and time zero** 5](#_Toc100919974)

[**Figure S2: Selection bias in landmark studies** 5](file:////Users/maryquinn/Documents/Work/HSPH%20PhD/NVAS-DPT/Vaccine%20Submission/Supplementary%20material-4152022.docx#_Toc100919975)

[**Figure S3: Selection bias in the Jensen landmark approach.** 6](file:////Users/maryquinn/Documents/Work/HSPH%20PhD/NVAS-DPT/Vaccine%20Submission/Supplementary%20material-4152022.docx#_Toc100919976)

[**Table S2: Three proposed methods and exposure classification** 7](#_Toc100919977)

[**Table S3: Bounds Sensitivity Analysis Assumptions** 9](#_Toc100919978)

[Data Description 10](#_Toc100919979)

[**Table S4: Ghana descriptive statistics by vitamin A supplementation** 10](#_Toc100919980)

[**Table S5: Tanzania descriptive statistics by vitamin A supplementation** 11](#_Toc100919981)

[**Table S6: Ghana descriptive statistics by BCG vaccination** 12](#_Toc100919982)

[**Table S7: Ghana descriptive statistics by DTP vaccination** 13](#_Toc100919983)

[**Table S8: Tanzania descriptive statistics by BCG vaccination** 14](#_Toc100919984)

[**Table S9: Tanzania descriptive statistics by DTP vaccination** 15](#_Toc100919985)

[**Figure S4: Flow chart of included data from Ghana trial** 16](#_Toc100919986)

[**Figure S5: Flow chart of included data from Tanzania trial** 17](#_Toc100919987)

[Bounds Analysis for Vaccination 18](#_Toc100919988)

[**Table S10: Sensitivity Analysis - Missingness Bounds for the association of BCG vaccination with infant mortality^a^** 18](#_Toc100919989)

[**Table S11: Sensitivity Analysis - Missingness Bounds for the association of DTP vaccination with infant mortality, from 30 to 365 days^a^** 20](#_Toc100919990)

[Combined Estimates 21](#_Toc100919991)

[**Table S12: Combined country estimates for the adjusted effect of Vitamin A Supplementation on Infant Mortality, stratified by BCG Vaccination Status and Infant Sex^a^** 21](#_Toc100919992)

[**Figure S6a: Combined forest plot of the adjusted effect of vitamin A supplementation on infant mortality, stratified by BCG vaccination status^a^** 22](#_Toc100919993)

[**Figure S6b: Combined forest plot of the adjusted effect of vitamin A supplementation on infant mortality, stratified by BCG vaccination status and sex^a^** 23](#_Toc100919994)

[**Table S13: Combined country estimates for the adjusted effect of vitamin A supplementation on infant mortality, stratified by DTP vaccination status and infant sex^a^** 24](#_Toc100919995)

[Bounds Analysis for Neonatal Vitamin A – Vaccine Interaction 25](#_Toc100919996)

[**Table S14: Sensitivity Analysis - Bounds of the adjusted effect of vitamin A supplementation on infant mortality, stratified by BCG vaccination status for all infants^a^** 25](#_Toc100919997)

[**Table S15: Sensitivity Analysis - Bounds of the adjusted effect of vitamin A supplementation on infant mortality, stratified by BCG vaccination status for female infants^a^** 27](#_Toc100919998)

[**Table S16: Sensitivity Analysis - Bounds of the adjusted effect of vitamin A supplementation on infant mortality, stratified by BCG vaccination status for male infants^a^** 29](#_Toc100919999)

[**Table S17: Sensitivity Analysis - Bounds of the adjusted effect of vitamin A supplementation on infant mortality, stratified by DTP vaccination status for all infants^a^** 31](#_Toc100920000)

[**Table S18: Sensitivity Analysis - Bounds of the adjusted effect of vitamin A supplementation on infant mortality, stratified by DTP vaccination status for female infants^a^** 33](#_Toc100920001)

[**Table S19: Sensitivity Analysis - Bounds of the adjusted effect of vitamin A supplementation on infant mortality, stratified by DTP vaccination status for male infants^a^** 35](#_Toc100920002)

[Effect modification by previous BCG 37](#_Toc100920003)

[**Table S20: Effect modification by previous BCG on the association of DTP and survival** 37](#_Toc100920004)

[Theorized Mechanisms of Action 38](#_Toc100920005)

[**Table S21: Theorized mechanisms of action for the nonspecific BCG and DTP effects and interactions** 38](#_Toc100920006)

# Methods

## **Method Selection and Bias**

There are four primary sources of bias in the types of vaccination analyses that we consider in the study: immortal time bias, selection bias, differential misclassification of exposures, and confounding (sometimes referred to as frailty bias). In past literature, two methods have been proposed to analyze these types of data to minimize the influence of these four biases: the retrospective updating approach and the landmark approach. We can define these two approaches based on the 2007 paper by Jensen in *Tropical Medicine & International* *Health*.(1) Building on these methods, we have used a hybrid approach in our paper.

The **retrospective updating approach,** as described by Jensen and colleagues (1), includes vaccination status as a time varying variable allowed to update at the time of vaccination. This approach assumes that all missing vaccination data can be classified as “unvaccinated time.” As an alternative, the **Jenson landmark approach** was proposed to avoid immortal time bias and differential misclassification of exposures inherent to the retrospective updating approach. This method sets time zero at the first visit where a vaccine card was seen. This type of analysis may introduce additional exposure misclassification since exposure status is only allowed to vary at the time of the visit following the change in exposure status. This is intended to change the type of misclassification from differential to nondifferential. It also excludes a large number of individuals who had a missed visit. **Our hybrid approach** aims to include the maximum amount of information and affords the ability to conduct sensitivity analyses that estimate the bounds for missing vaccination data. Time zero is set at vaccine eligibility the vaccination is allowed to update at the true time of vaccination. Table S1 summarizes the similarities and differences between our approach, and these two approaches described above, as discussed by Jensen and colleagues.

In the following sections, we compare our approach against those described by Jensen and colleagues with respect to how they address immortal time bias, selection bias, differential misclassification, and confounding. While all three methods will produce biased estimates due to the tradeoffs between immortal time bias, selection bias, and misclassification: however, our approach allows the contribution of some of these biases to be estimated using bounds. We preferred this approach to the unclear direction and contribution of bias in the other two approaches.

## **Table S1: Description of three proposed methods**

|  | **Method 1: Retrospective updating** | **Method 2: Jensen landmark approach** | **Method 3: Our hybrid approach** |
| --- | --- | --- | --- |
| Start of follow-up | At birth | At the first follow-up visit where the vaccine is observed after the participant is eligible for the vaccine | At vaccine eligibility |
| Type of exposure | Vaccination status (yes/no) is a time-varying variable based on date of vaccination | Vaccination status (yes/no) is a time-fixed variable based on dates the vaccine card was observed | Vaccine status (yes/no) is a time-varying variable based on the date of vaccination |
| Definition for being unvaccinated | No vaccine card observed or no date of vaccination | Vaccine card observed and no date of vaccination | Vaccine card observed with no vaccine listed or guardian interview confirmed unvaccinated |
| Definition of being vaccinated | Vaccine card observed with date of vaccination | Vaccine card observed and vaccine documented | Vaccine card observed or guardian interview confirmed vaccination |
| Dealing with missing exposure data | Time periods with missing vaccination data assumed to be unvaccinated | Time periods when no vaccination card was observed or vaccination data missing excluded | Missing data is excluded but addressed with sensitivity analysis. |

*Tradeoff between Immortal time bias & selection bias*

Immortal time bias occurs when a study design defines the exposure in a way that artificially creates a period of time during which a death cannot occur (i.e. the participant is “immortal” during this time period).(2) This type of bias has also been referred to as survival bias (3) or guarantee-time bias.(4) Hernan et al. describe how this bias arises when there is misalignment between the time when eligibility criteria are assessed (e.g. being born into a birth cohort), when treatment strategies are assigned (e.g. vaccination status), and start of follow-up (i.e. time zero). Strategies like the landmark approach can avoid immortal time bias; however, they do not inherently address misalignments between these three elements (eligibility, treatment assignment and time zero), and thus can cause selection bias. Table S2 shows how each type of analysis excludes different individuals from the analysis. These exclusions each introduce a risk of selection bias.

In our study, we are concerned about immortal time bias and selection bias in the time between “vaccine eligibility” and the actual time of vaccination (Figure 1). If we think of our analysis in the context of a target trial,(5) some individuals who would have received the vaccine later, for example at 6 months of age for DTP, may die before actually receiving the treatment. In the target trial, this individual could have been randomly allocated to the vaccinated group, but our observational analysis would falsely allocate them in the unvaccinated group.

## **Figure S1: Immortal time bias and alignment of eligibility, treatment, and time zero**


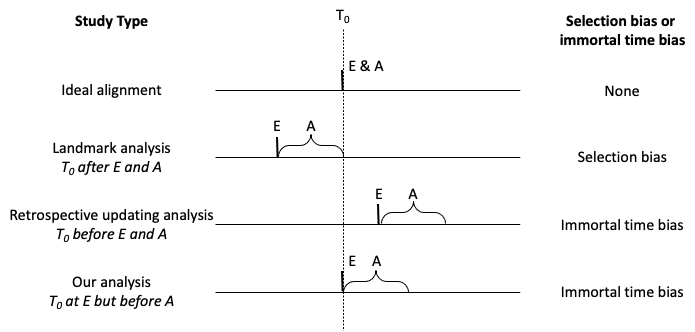


*The alignment of eligibility (E), treatment assignment (A), and time zero (T_0_) in the three proposed study types. Misalignment of E, A, and T_0_ can result in immortal time bias when T_0_ is set before assignment. However, setting T_0_ after eligibility and treatment assignment restricts to those still under follow up at T_0_ and can result in selection bias. Figure adapted from Hernan and colleagues.(5)*


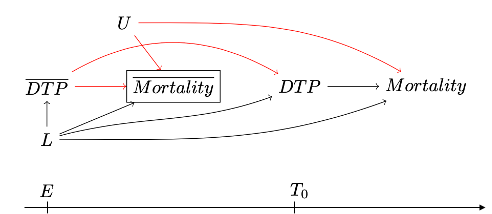
Several methods have been proposed to address immortal time bias. Jensen and colleagues (1) suggest setting a landmark (time zero) later during follow up to creates an estimate that would be less biased by the inclusion of immortal time. However, landmark analyses also induce selection bias because only those surviving to the landmark are included in the analysis.(5) This type of selection bias is shown in directed acyclic graph (DAG) (Figure S2). In the DAG, selection occurs on mortality prior to the landmark date, since earlier mortality in infancy would result in exclusion from the analysis. If we are trying to accurately measure the effect of DTP on mortality, the result will be biased by any unmeasured confounders, U, such as infant health status, that can open a pathway between survival earlier in life and later in life. This results in collider stratification bias for the effect of later DTP on later mortality.

## **Figure S2: Selection bias in landmark studies**

Selection bias in landmark studies. L refers to measured confounders. U refers to other unmeasured confounders, like infant health status. DTP and mortality that occur before the landmark date are denoted as $\bar{DTP}$ and $\bar{Mortality}.$ The time of eligibility for vaccination is shown as E and time zero is shown as $T_{o}.$ The biasing pathway for the effect of DTP on Mortality is shown in red.


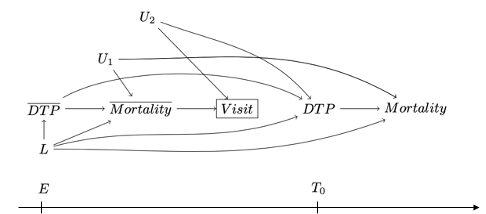
In the Jensen landmark approach, the landmark is set when the vaccine card is observed. This approach assumes that missed visits occur at random. However, an additional source selection bias can occur in this setting. Any unmeasured common cause of missing the study visit and missing the vaccine will induce additional selection bias in this landmarked analysis. This is shown in the additional biasing pathway through U_2_ in Figure S3.

## **Figure S3: Selection bias in the Jensen landmark approach.**

L refers to measured confounders. U_1_ refers to other unmeasured confounders, like infant health status which are a common cause of early life survival and later survival. U_1_ refers to other unmeasured confounders, like economic or social circumstances that are a common cause of missing a vaccine and missing a study visit. DTP and mortality that occur before the landmark date are denoted as $\bar{DTP}$ and $\bar{Mortality}.$ The time of eligibility for vaccination is shown as E and time zero is shown as $T_{o}.$

*Differential Misclassification of Exposures*

Misclassification in vaccine studies occurs when the true vaccination status at time t of the child is not accurately classified in the data. This exposure misclassification causes bias when it occurs differentially by the outcome of the study participant. In our analysis, participants are more likely to be missing exposure information (vaccine status), and thus be misclassified, when the participant dies. This happens because the child’s health card (the source of verified vaccine status information) was not always available after the child’s death. Furthermore, if we assume that a child is “unvaccinated” when the child’s health card is missing after death, this could result in more misclassification if participants with missing data were more likely to be vaccinated.

We explore how each of the three methods handle misclassification given ten ‘information’ scenarios observed in the data (Table S1 and Figures S3-S4). In scenarios 1 and 2, all three approaches correctly classify the data. In scenario 3, retrospective updating and our hybrid approach correctly classify the data, but the landmark approach misclassifies data between the time of the first and second visit.

In all three methods, scenario 4 is incorrectly classified where the vaccine is given between the last visit and death and the vaccine card is not available after death. The retrospective updating approach does not address this potential source of bias. The Jensen approach tries to correct for it by misclassifying data in the opposite direction (scenario 3). Our approach did not add intentional misclassification in the opposite direction, and the misclassification from scenario 4 may be a source of bias in our results.

The retrospective updating approach further misclassifies scenarios 7 and 8 by incorrectly assigning participants with missing information to the “unvaccinated” group. Both our analysis and the Jenson approach exclude these two cases. Because the missing data in this type of vaccine study is likely missing not at random, we used a bounds analysis to investigate whether the missing data was biasing our estimates. In our approach, we included a sensitivity analysis to test whether our approach was robust to extreme values of for missing data. This type of sensitivity analysis cannot be performed using the Jensen approach and excluding these scenarios may bias the final estimates. In the Jensen approach, scenario 6 is also excluded; however, our hybrid analysis includes this case and classifies the time correctly.

*Confounding*

In vaccination studies, confounding is sometimes referred to as frailty bias, due to unmeasured confounding by infant health status. In these studies, we are concerned with unmeasured confounding by access to care, in addition to infant health and other factors. All three proposed methods are equally likely to be at risk of confounding.

## **Table S2: Three proposed methods and exposure classification**

|  | Information scenarios: | Method 1: Retrospective updating approach | Method 2:  Jensen Landmark approach | Method 3:  Our hybrid approach with sensitivity analysis |
| --- | --- | --- | --- | --- |
| 1 | 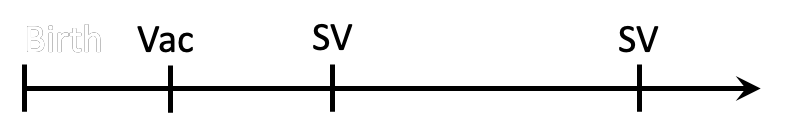 | Correctly classified | Correctly classified | Correctly classified |
| 2 | 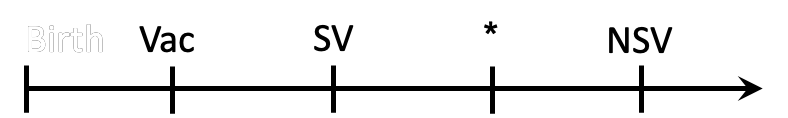 | Correctly classified | Correctly classified | Correctly classified |
| 3 | 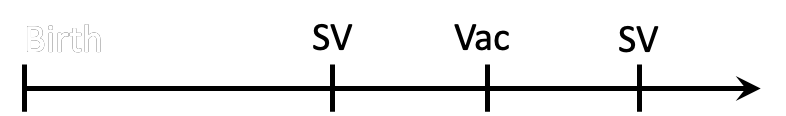 | Correctly classified | Misclassifies data between vaccine and second visit | Correctly classified |
| 4 | 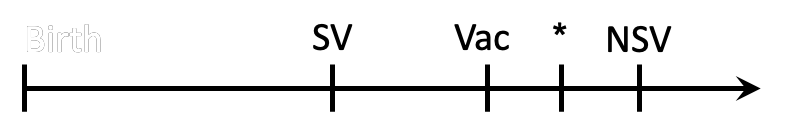 | Misclassifies data between vaccination and death | Misclassifies data between vaccination and death | Misclassifies data between vaccination and death |
| 5 | 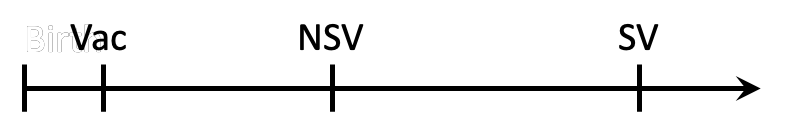 | Correctly classified | Excludes from analysis | Correctly classifies |
| 6 | 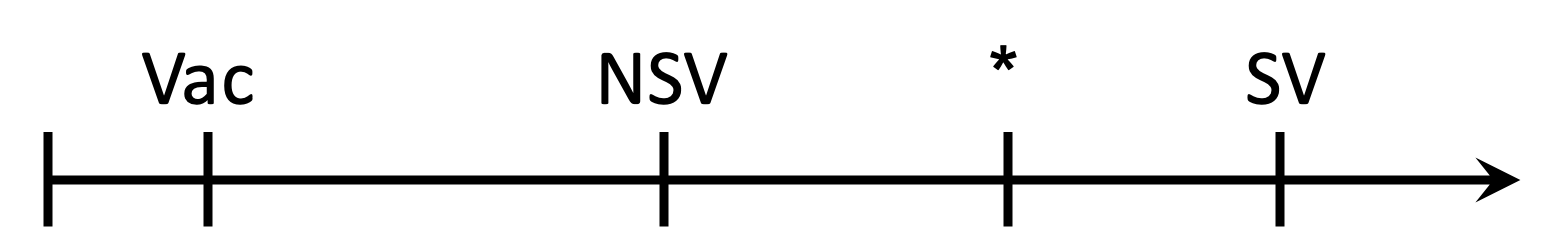 | Correctly classifies | Excludes from analysis | Correctly classifies |
| 7 | 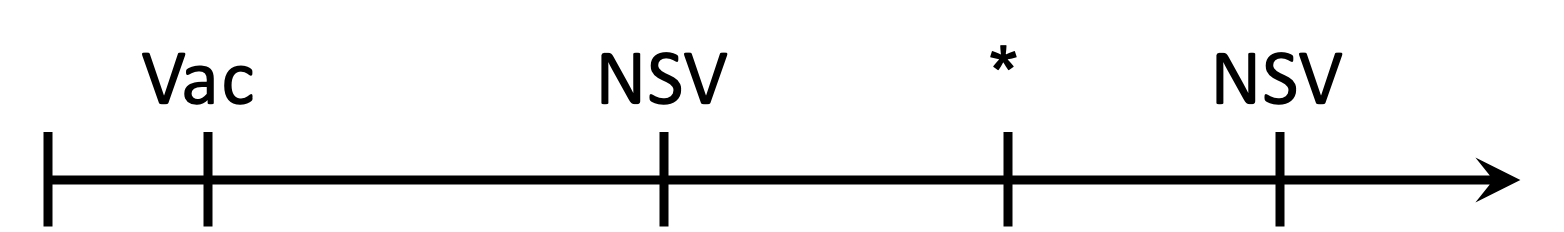 | Correctly classifies | Excludes from analysis | Main analysis excludes but impact assessed through sensitivity analysis |
| 8 | 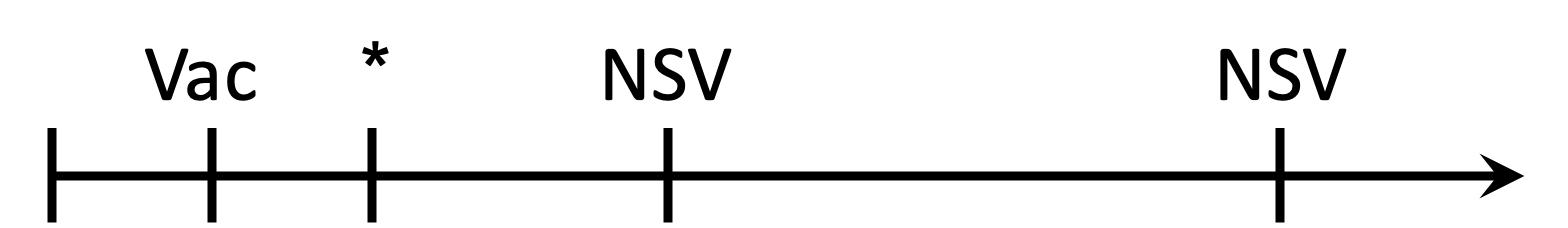 | Misclassifies data between vaccination and death | Excludes from analysis | Main analysis excludes but impact assessed through sensitivity analysis |
| 9 | 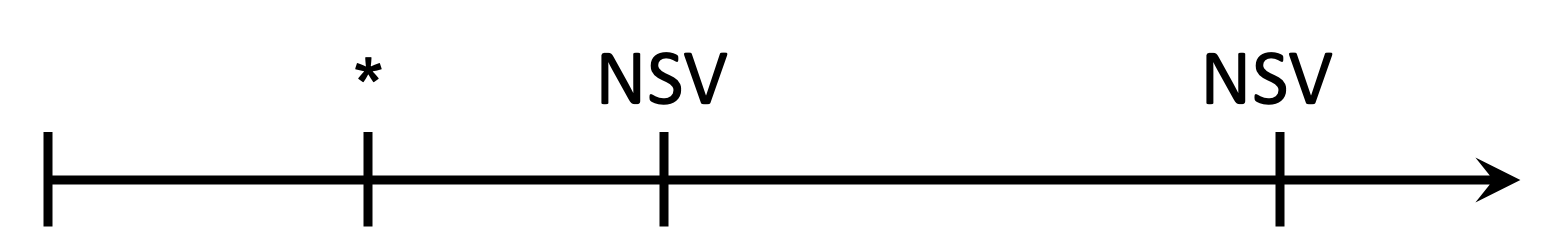 | Misclassifies data between vaccination and death | Excludes from analysis | Main analysis excludes but impact assessed through sensitivity analysis |
|  | *Vac* Point in time that the vaccine was received  *NSV* No vaccine card seen at visit  *SV* Vaccine card seen at visit  * Point in time of death |  |  |  |

*Cases based on the description of the landmarking method proposed in Jensen et al., 2007.*

**Figure S4: Description of misclassification and exclusion from different analysis approaches**

|  | Information scenarios | Method 1:  Retrospective updating approach | Method 2:  Jensen landmark approach | Method 3:  Our hybrid approach: |
| --- | --- | --- | --- | --- |
| 1 | 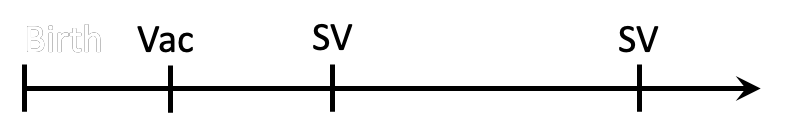 | 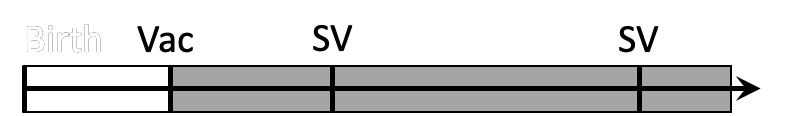 | 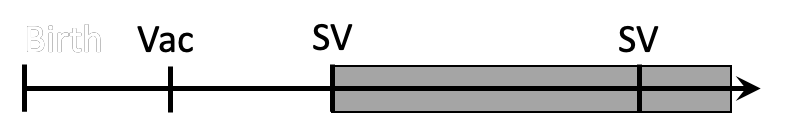 | 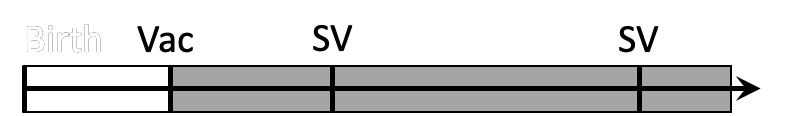 |
| 2 | 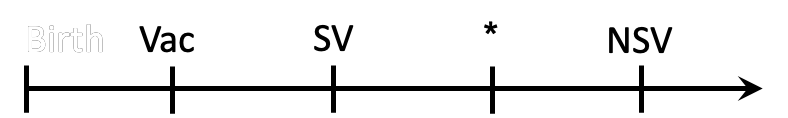 | 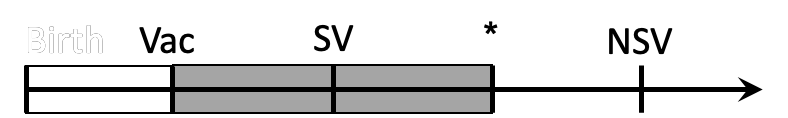 | 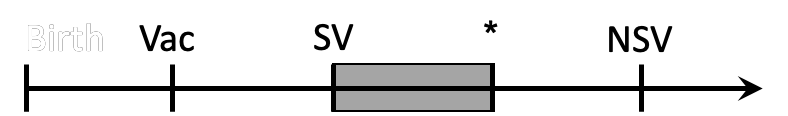 | 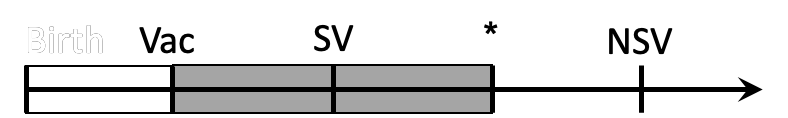 |
| 3 | 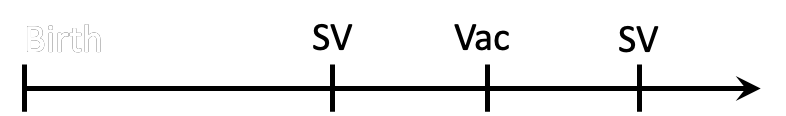 | 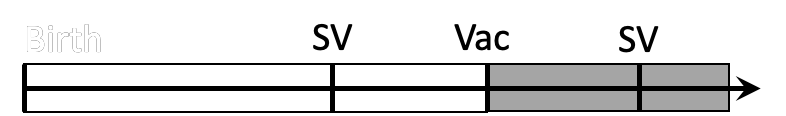 | 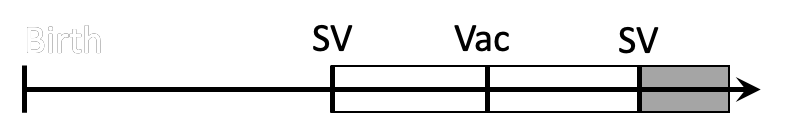 | 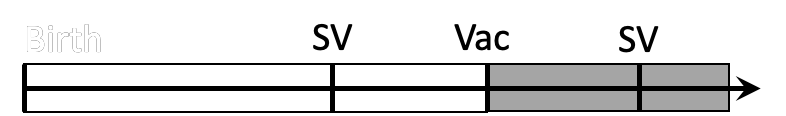 |
| 4 | 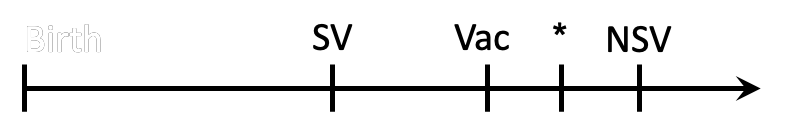 | 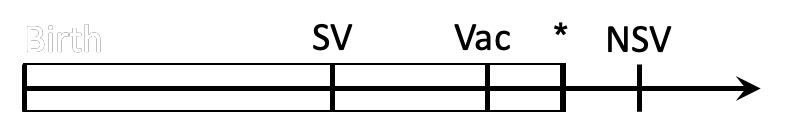 | 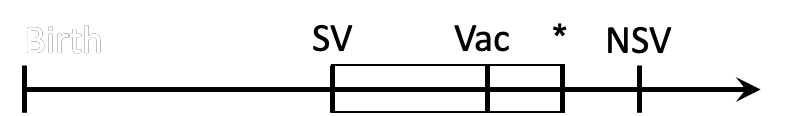 | 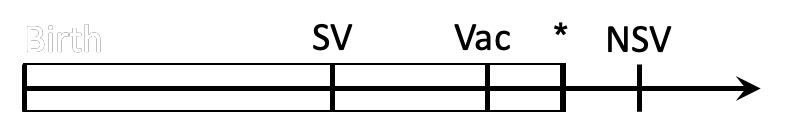 |
| 5 | 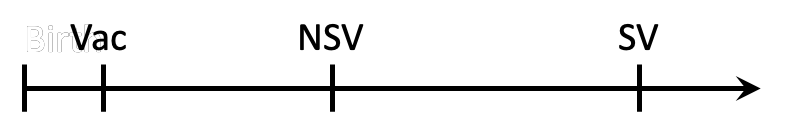 | 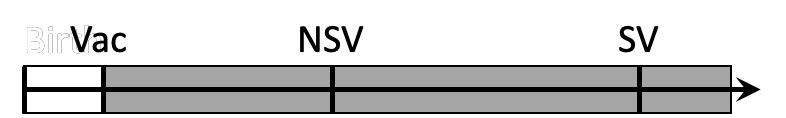 | 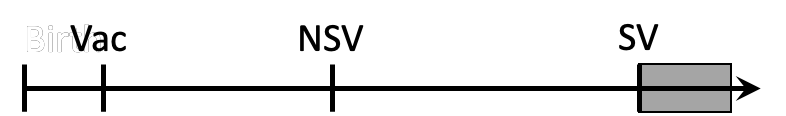 | 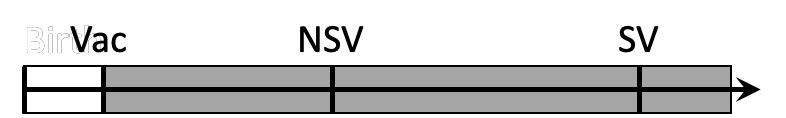 |
| 6 | 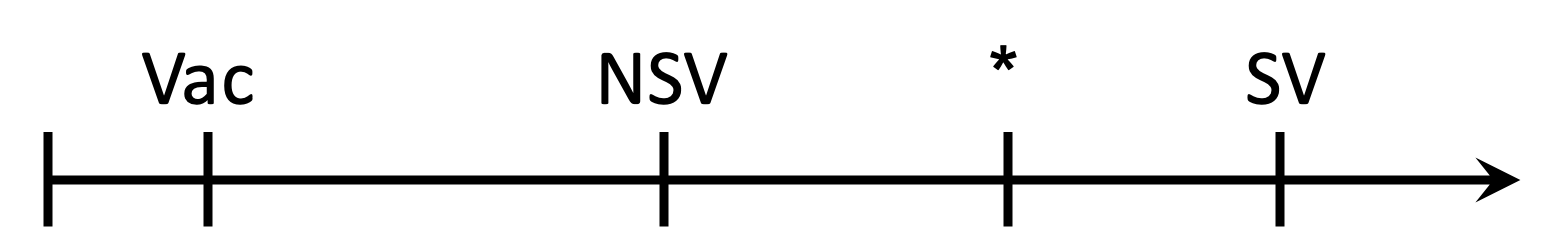 | 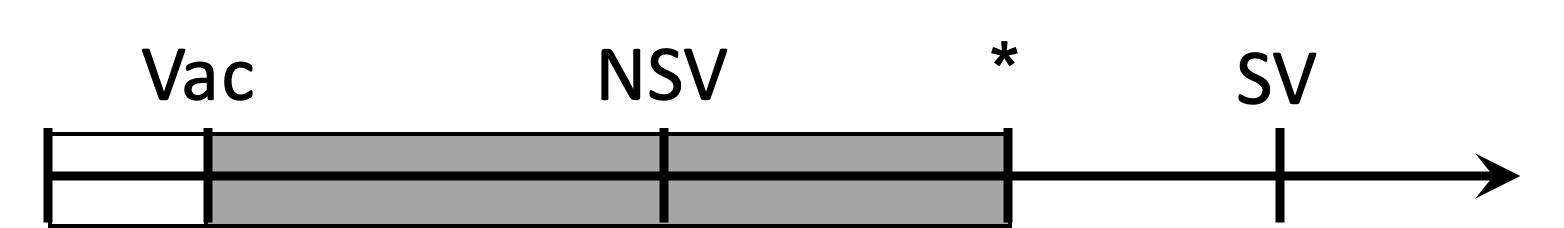 | 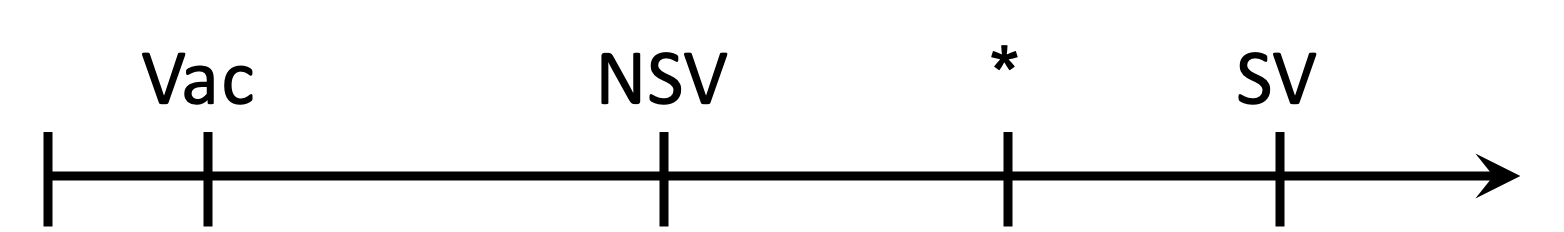 | 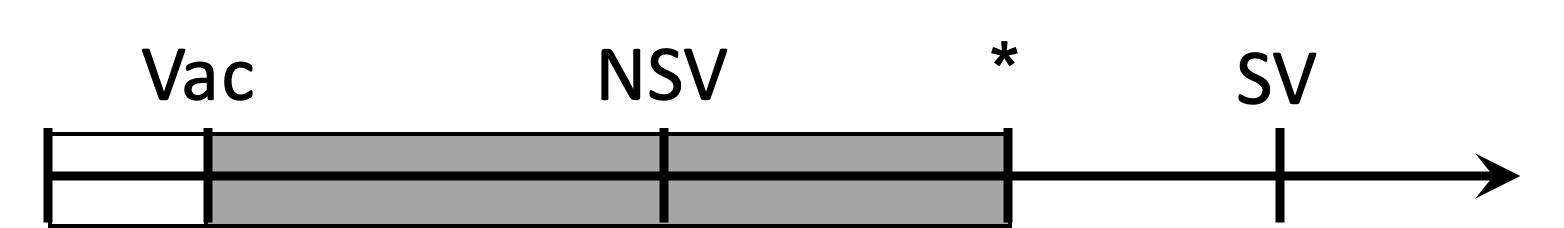 |
| 7 | 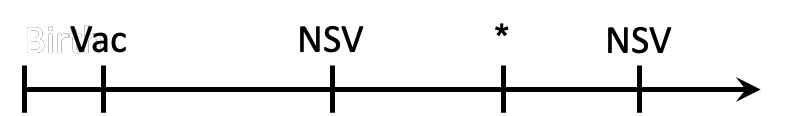 | 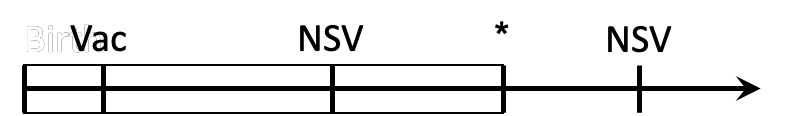 | 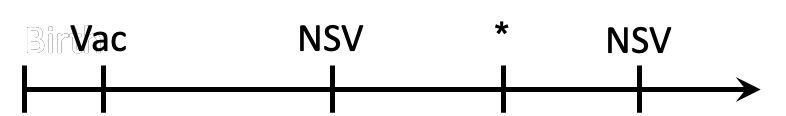 | 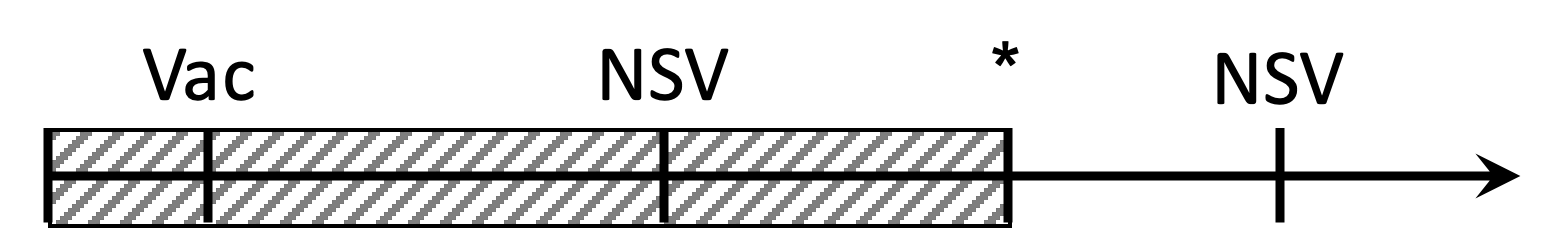 |
| 8 | 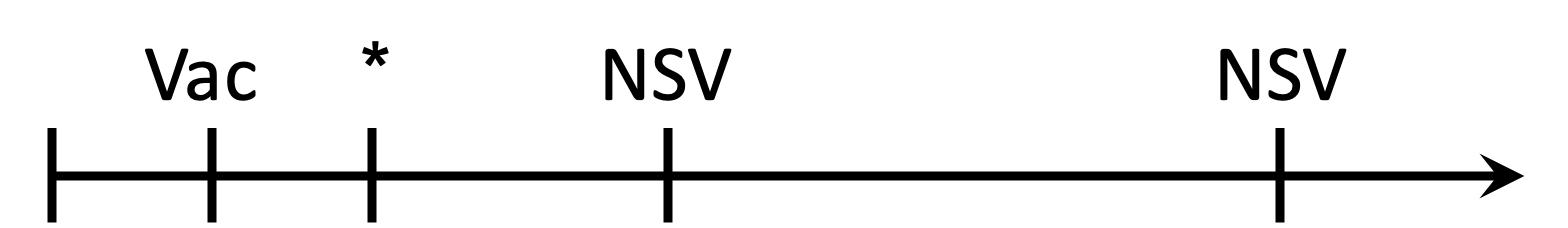 | 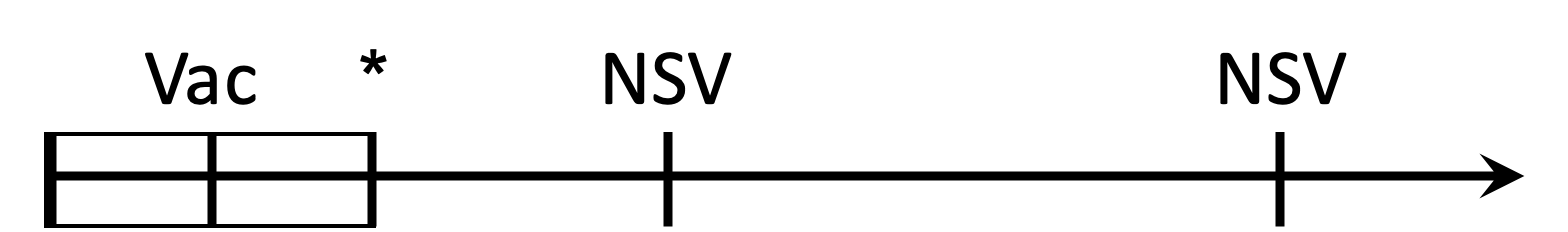 | 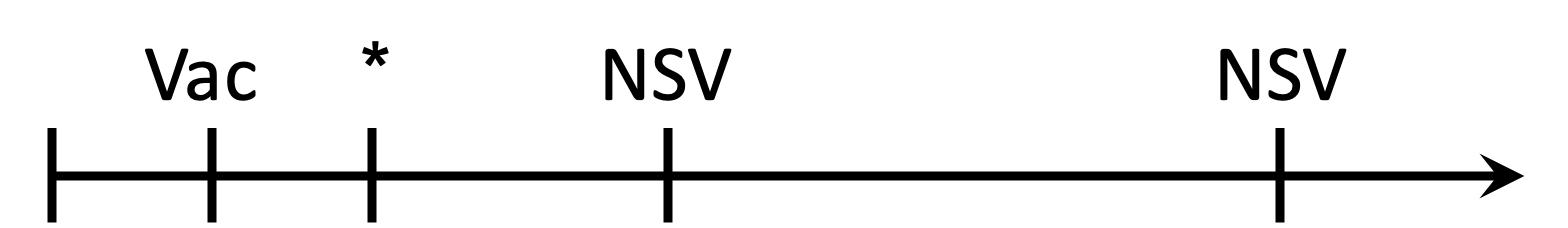 | 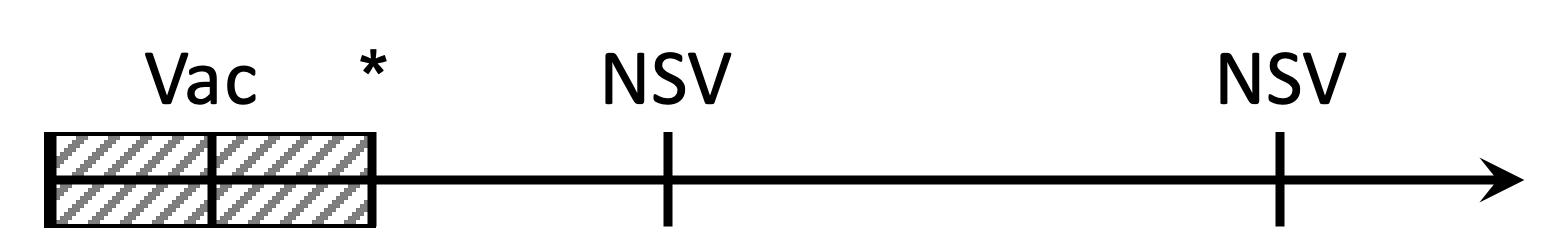 |
| 9 | 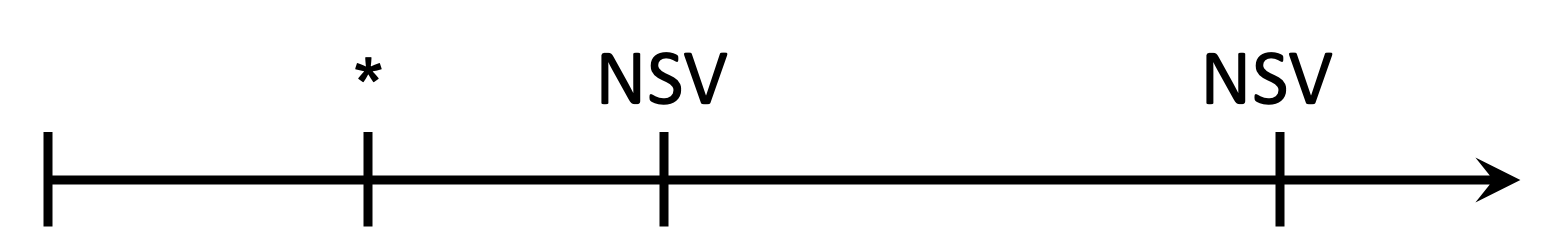 | 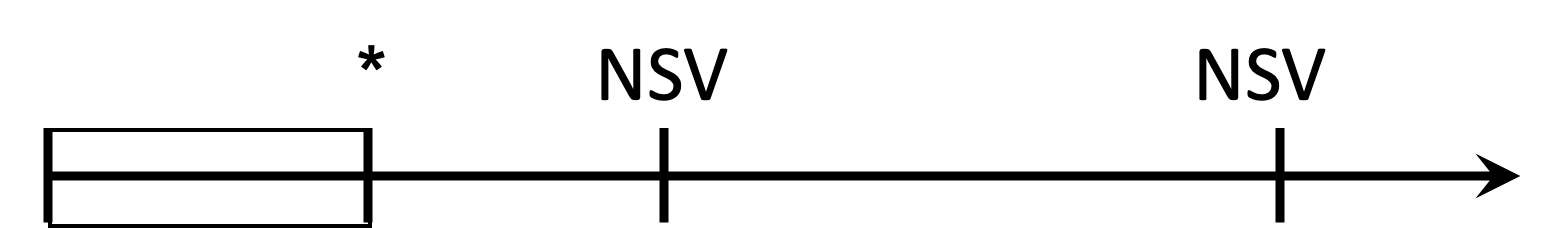 | 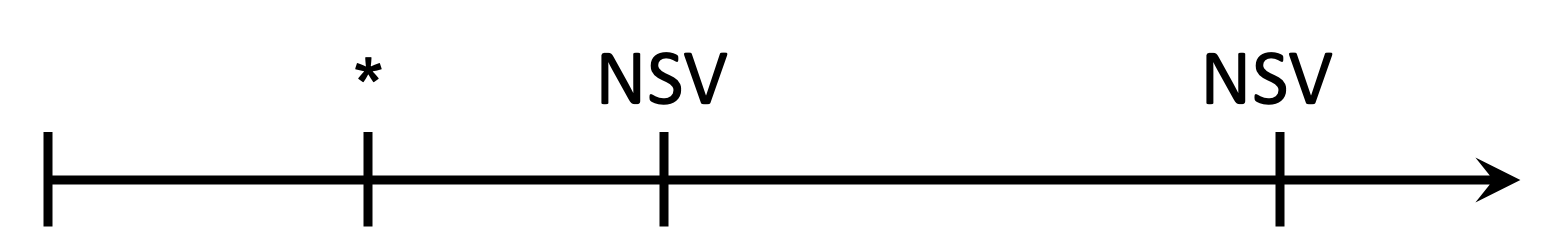 | 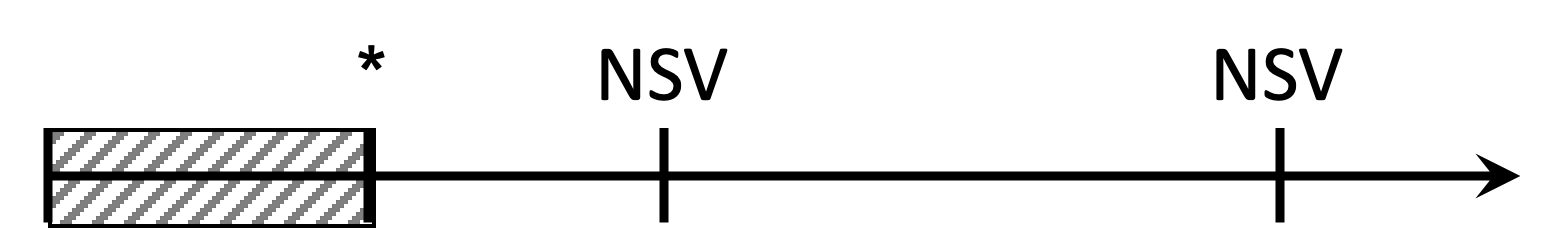 |
|  | 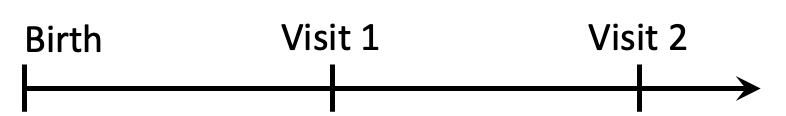 | 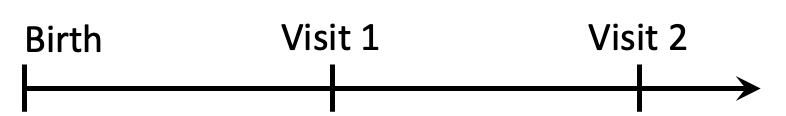 | 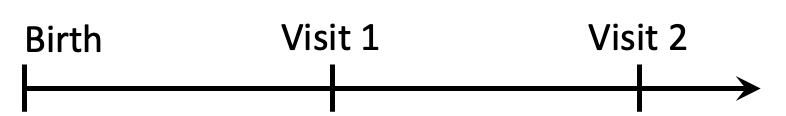 | 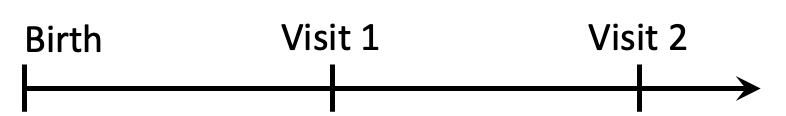 |

*Vac* Point in time that the vaccine was received

*NSV* No vaccine card seen at visit

*SV* Vaccine card seen at visit

* Point in time of death

Unvaccinated time


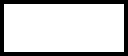


Vaccinated time


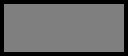


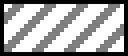
 Time addressed in sensitivity analysis

*Cases are based on the description of the landmarking approach described by Jensen et al 2007. In the landmark approach all person-time from scenarios 6-9 are excluded due to missing vaccine information. Our primary analysis excludes scenarios 7-9. However, because exclusion of these cases may cause selection bias, the contribution of this bias is analyzed in supplemental tables S10-S15.*

## **Table S3: Bounds Sensitivity Analysis Assumptions**

| **Missingness assumptions** | **For each participant who is vaccinated, but missing the date of vaccination:** | **For each participant whose vaccination status is not known:** |
| --- | --- | --- |
| **Scenario 1:**  All participants missing data **vaccinated at baseline**:   - 30 days for DTP - 0 days for BCG | Assume:  30 Day Vaccination for DTP  0 Day Vaccination for BCG | Assume:  30 Day Vaccination for DTP  0 Day Vaccination for BCG |
| **Scenario 2:**  All participants missing data **vaccinated at the mode**:   - 31 days for DTP - 1 days for BCG | If total follow up < the mode, assume  total follow up date as vaccination date.  If total follow up ≥ the mode, assume the mode as vaccination date. | If total follow up < mode, assume they are unvaccinated.  If total follow up ≥ mode, assume the mode as vaccination date. |
| **Scenario 3:**  All participants missing data **unvaccinated** | If total follow up < the mode, assume the total follow up date as vaccination date.  If total follow up ≥ the mode, assume the mode as vaccination date. | Assume all are unvaccinated. |

# Data Description

## **Table S4: Ghana descriptive statistics by vitamin A supplementation**

| Covariate |  | Vitamin A  n=11,474 | | Placebo  n=11,481 | |
| --- | --- | --- | --- | --- | --- |
|  |  | n | (%) | n | (%) |
| Age at BCG vaccination, mean days ±SD | | 18 | ± 29 | 19 | ± 28 |
| Age at DTP vaccination, mean days ±SD | | 61 | ± 21 | 61 | ± 21 |
| Birthweight | <1.5kg | 56 | (0.5%) | 61 | (0.5%) |
|  | 1.50-1.99kg | 230 | (2.0%) | 214 | (1.9%) |
|  | 2.00-2.49kg | 1,523 | (13.3%) | 1,508 | (13.1%) |
|  | >=2.5kg | 9,665 | (84.2%) | 9,698 | (84.5%) |
| Head of Household | Father of baby | 8,920 | (77.7%) | 9,022 | (78.6%) |
|  | Grandfather | 591 | (5.2%) | 577 | (5%) |
|  | Grandmother | 1,604 | (14%) | 1,518 | (13.2%) |
|  | Mother of baby | 203 | (1.8%) | 224 | (2.0%) |
|  | Other | 156 | (1.4%) | 140 | (1.2%) |
| Household Religion | Christian | 7,954 | (69.3%) | 8,025 | (69.9%) |
|  | Muslim | 2,783 | (24.3%) | 2,728 | (23.8%) |
|  | None | 565 | (4.9%) | 542 | (4.7%) |
|  | Trad African | 172 | (1.5%) | 186 | (1.6%) |
| Maternal age group | <20 | 2,079 | (18.1%) | 2,102 | (18.3%) |
|  | 20-24 | 3,012 | (26.3%) | 2,944 | (25.6%) |
|  | 25-29 | 3,221 | (28.1%) | 3,206 | (27.9%) |
|  | 30-34 | 1,844 | (16.1%) | 1,896 | (16.5%) |
|  | 35-39 | 1,065 | (9.3%) | 1,072 | (9.3%) |
|  | 40+ | 253 | (2.2%) | 261 | (2.3%) |
| Maternal education | None | 3,579 | (31.2%) | 3,562 | (31%) |
|  | Primary | 2,111 | (18.4%) | 2,125 | (18.5%) |
|  | Secondary | 5,577 | (48.6%) | 5,595 | (48.7%) |
|  | Post-Secondary | 207 | (1.8%) | 199 | (1.7%) |
| Multiple or Singleton Birth | Multiple | 438 | (3.8%) | 409 | (3.6%) |
|  | Single | 11,036 | (96.2%) | 11,072 | (96.4%) |
| Number of living children in household | 1 | 3,376 | (29.4%) | 3,356 | (29.2%) |
|  | 2 | 2,567 | (22.4%) | 2,551 | (22.2%) |
|  | ≥3 | 5,531 | (48.2%) | 5,574 | (48.5%) |
| Number of children in household who have died | 0 | 9,283 | (80.9%) | 9,271 | (80.8%) |
|  | 1 | 1,635 | (14.2%) | 1,669 | (14.5%) |
|  | 2 | 418 | (3.6%) | 402 | (3.5%) |
|  | ≥3 | 138 | (1.2%) | 139 | (1.2%) |
| Place of birth | Compound | 2,659 | (23.2%) | 2,627 | (22.9%) |
|  | Facility | 8,770 | (76.4%) | 8,811 | (76.7%) |
|  | Other | 45 | (0.4%) | 43 | (0.4%) |
| Site ID | 1 | 2,425 | (21.1%) | 2,427 | (21.1%) |
|  | 2 | 2,784 | (24.3%) | 2,782 | (24.2%) |
|  | 3 | 4,172 | (36.4%) | 4,176 | (36.4%) |
|  | 4 | 2,093 | (18.2%) | 2,096 | (18.3%) |
| Wealth Quintile | 1 (lowest) | 2,283 | (19.9%) | 2,308 | (20.1%) |
|  | 2 | 2,306 | (20.1%) | 2,285 | (19.9%) |
|  | 3 | 2,294 | (20.0%) | 2,298 | (20.0%) |
|  | 4 | 2,270 | (19.8%) | 2,320 | (20.2%) |
|  | 5 (highest) | 2,321 | (20.2%) | 2,270 | (19.8%) |
| Type of delivery | Caesarean | 941 | (8.2%) | 934 | (8.1%) |
|  | Vaginal | 10,532 | (91.8%) | 10,547 | (91.9%) |
| Maternal Vitamin A Megadose | No | 5,593 | (48.7%) | 5,698 | (49.6%) |
|  | Yes | 5,879 | (51.2%) | 5,777 | (50.3%) |
|  | Not Known | 2 | (0.0%) | 6 | (0.1%) |

## **Table S5: Tanzania descriptive statistics by vitamin A supplementation**

| Covariate |  | Vitamin A  n=15,995 | | Placebo  n=16,004 | |
| --- | --- | --- | --- | --- | --- |
|  |  | n | (%) | n | (%) |
| Age at BCG vaccination, mean days ± SD | | 10 | ± 22 | 10 | ± 22 |
| Age at DTP vaccination, mean days ± SD | | 43 | ± 20 | 43 | ± 20 |
| Birthweight | <1.5kg | 31 | (0.2%) | 34 | (0.2%) |
|  | 1.50-1.99kg | 111 | (0.7%) | 99 | (0.6%) |
|  | 2.00-2.49kg | 1,766 | (11.0%) | 1,841 | (11.4%) |
|  | >=2.5kg | 14,087 | (88.1%) | 14,030 | (87.7%) |
| Head of Household | Father of baby | 11,823 | (73.9%) | 12,008 | (75.0%) |
|  | Grandfather | 1,123 | (7.0%) | 1,044 | (6.5%) |
|  | Grandmother | 773 | (4.8%) | 759 | (4.7%) |
|  | Mother of baby | 846 | (5.3%) | 821 | (5.1%) |
|  | Other | 299 | (1.9%) | 260 | (1.6%) |
|  | Missing | 1,131 | (7.1%) | 1,112 | (6.9%) |
| Household Religion | Christian | 5,902 | (36.9%) | 5,921 | (37.0%) |
|  | Muslim | 4,405 | (27.5%) | 4,360 | (27.2%) |
|  | None | 350 | (2.2%) | 369 | (2.3%) |
|  | Traditional African & Other | 44 | (0.2%) | 46 | (0.3%) |
|  | Missing | 5,294 | (33.1%) | 5,308 | (33.2%) |
| Maternal age group | <20 | 2,198 | (13.7%) | 2,141 | (13.4%) |
|  | 20-24 | 4,497 | (28.1%) | 4,524 | (28.3%) |
|  | 25-29 | 4,265 | (26.7%) | 4,278 | (26.7%) |
|  | 30-34 | 2,704 | (16.9%) | 2,704 | (16.9%) |
|  | 35-39 | 1,148 | (7.2%) | 1,229 | (7.7%) |
|  | 40+ | 296 | (1.9%) | 275 | (1.7%) |
|  | Missing | 887 | (5.5%) | 853 | (5.3%) |
| Maternal education | None | 1,340 | (8.4%) | 1,316 | (8.2%) |
|  | Primary | 12,176 | (76.1%) | 12,252 | (76.6%) |
|  | Secondary or more | 1,680 | (10.5%) | 1,653 | (10.3%) |
|  | Missing | 799 | (5.0%) | 783 | (4.9%) |
| Multiple or Singleton Birth | Multiple | 539 | (3.4%) | 569 | (3.6%) |
|  | Single | 15,456 | (96.6%) | 15,435 | (96.4%) |
| Number of living children in household | 1 | 3,424 | (21.4%) | 3,398 | (21.2%) |
|  | 2 | 4,397 | (27.5%) | 4,540 | (28.4%) |
|  | ≥3 | 7,335 | (45.9%) | 7,235 | (45.1%) |
|  | Missing | 839 | (5.2%) | 831 | (5.2%) |
| Number of children who have died in household | 0 | 12,803 | (80.0%) | 12,726 | (79.5%) |
|  | 1 | 1,020 | (6.4%) | 1,083 | (6.8%) |
|  | 2 | 413 | (2.6%) | 425 | (2.7%) |
|  | ≥3 | 405 | (2.5%) | 426 | (2.7%) |
|  | Missing | 1,354 | (8.5%) | 1,344 | (8.4%) |
| Place of birth | Home | 1,321 | (8.3%) | 1,332 | (8.3%) |
|  | Facility | 13,894 | (86.9%) | 13,882 | (86.7%) |
|  | Other | 551 | (3.4%) | 544 | (3.4%) |
|  | Missing | 229 | (1.4%) | 246 | (1.5%) |
| Site ID | Dar Es Salaam | 5,939 | (37.1%) | 5,956 | (37.2%) |
|  | Ifakara | 10,056 | (62.9%) | 10,048 | (62.8%) |
| Wealth Quintile | 1 (lowest) | 2,943 | (18.4%) | 3,029 | (18.9%) |
|  | 2 | 3,866 | (24.2%) | 3,801 | (23.8%) |
|  | 3 | 1,541 | (9.6%) | 1,511 | (9.4%) |
|  | 4 | 4,913 | (30.7%) | 4,904 | (30.6%) |
|  | 5 (highest) | 2,732 | (17.1%) | 2,759 | (17.2%) |
| Type of delivery | Caesarean | 935 | (5.8%) | 928 | (5.8%) |
|  | Vaginal | 15,060 | (94.2%) | 15,076 | (94.2%) |
| Maternal Vitamin A Megadose | No | 5,132 | (32.1%) | 5,161 | (32.2%) |
|  | Yes | 10,604 | (66.3%) | 10,560 | (66.0%) |
|  | Missing | 259 | (1.6%) | 283 | (1.8%) |

## **Table S6: Ghana descriptive statistics by BCG vaccination**

| Covariate |  | On Time  (≤ 1 day)  n=4,327 | Late  (> 1 day)  n=17,667 | Never  n=348 | Missing  n=613 |
| --- | --- | --- | --- | --- | --- |
|  |  | n (%) | n (%) | n (%) | n (%) |
| Birthweight | <1.5kg | 7 (0.2%) | 72 (0.4%) | 10 (2.9%) | 28 (4.6%) |
|  | 1.50-1.99kg | 66 (1.5%) | 313 (1.8%) | 22 (6.3%) | 43 (7.0%) |
|  | 2.00-2.49kg | 529 (12.2%) | 2,341 (13.3%) | 67 (19.3%) | 94 (15.3%) |
|  | >=2.5kg | 3,725 (86.1%) | 14,941 (84.6%) | 249 (71.6%) | 448 (73.1%) |
| Head of Household | Father of baby | 3,505 (81.0%) | 13,728 (77.7%) | 268 (77.0%) | 441 (71.9%) |
|  | Grandfather | 164 (3.8%) | 931 (5.3%) | 25 (7.2%) | 48 (7.8%) |
|  | Grandmother | 528 (12.2%) | 2,448 (13.9%) | 39 (11.2%) | 107 (17.5%) |
|  | Mother of baby | 84 (1.9%) | 337 (1.9%) | 5 (1.4%) | 1 (0.2%) |
|  | Other | 46 (1.1%) | 223 (1.3%) | 11 (3.2%) | 16 (2.6%) |
| Household Religion | Christian | 3,095 (71.5%) | 12,271 (69.5%) | 209 (60.1%) | 404 (65.9%) |
|  | Muslim | 1,058 (24.5%) | 4,184 (23.7%) | 112 (32.2%) | 157 (25.6%) |
|  | None | 132 (3.1%) | 917 (5.2%) | 17 (4.9%) | 41 (6.7%) |
|  | Trad African | 42 (1.0%) | 295 (1.7%) | 10 (2.9%) | 11 (1.8%) |
| Maternal age group | <20 | 742 (17.1%) | 3,202 (18.1%) | 89 (25.6%) | 148 (24.1%) |
|  | 20-24 | 1,123 (26.0%) | 4,587 (26.0%) | 74 (21.3%) | 172 (28.1%) |
|  | 25-29 | 1,254 (29.0%) | 4,929 (27.9%) | 88 (25.3%) | 156 (25.4%) |
|  | 30-34 | 698 (16.1%) | 2,902 (16.4%) | 56 (16.1%) | 84 (13.7%) |
|  | 35-39 | 417 (9.6%) | 1,652 (9.4%) | 30 (8.6%) | 38 (6.2%) |
|  | 40+ | 93 (2.1%) | 395 (2.2%) | 11 (3.2%) | 15 (2.4%) |
| Maternal education | None | 1,029 (23.8%) | 5,736 (32.5%) | 169 (48.6%) | 207 (33.8%) |
|  | Primary | 722 (16.7%) | 3,329 (18.8%) | 64 (18.4%) | 121 (19.7%) |
|  | Secondary | 2,469 (57.1%) | 8,314 (47.1%) | 115 (33.0%) | 274 (44.7%) |
|  | Post-Secondary | 107 (2.5%) | 288 (1.6%) | 0 (0%) | 11 (1.8%) |
| Multiple or Singleton Birth | Multiple | 161 (3.7%) | 625 (3.5%) | 22 (6.3%) | 39 (6.4%) |
|  | Single | 4,166 (96.3%) | 17,042 (96.5%) | 326 (93.7%) | 574 (93.6%) |
| Number of living children in household | 1 | 1,468 (33.9%) | 4,938 (28.0%) | 103 (29.6%) | 223 (36.4%) |
|  | 2 | 1,017 (23.5%) | 3,906 (22.1%) | 65 (18.7%) | 130 (21.2%) |
|  | ≥3 | 1,842 (42.6%) | 8,823 (49.9%) | 180 (51.7%) | 260 (42.4%) |
| Number of children in household who have died | 0 | 3,617 (83.6%) | 14,179 (80.3%) | 274 (78.7%) | 484 (79.0%) |
|  | 1 | 544 (12.6%) | 2,603 (14.7%) | 55 (15.8%) | 102 (16.6%) |
|  | 2 | 131 (3.0%) | 652 (3.7%) | 14 (4.0%) | 23 (3.8%) |
|  | ≥3 | 35 (0.8%) | 233 (1.3%) | 5 (1.4%) | 4 (0.7%) |
| Place of birth | Compound | 184 (4.3%) | 4,785 (27.1%) | 136 (39.1%) | 181 (29.5%) |
|  | Facility | 4,136 (95.6%) | 12,806 (72.5%) | 209 (60.1%) | 430 (70.1%) |
|  | Other | 7 (0.2%) | 76 (0.4%) | 3 (0.9%) | 2 (0.3%) |
| Site ID | 1 | 360 (8.3%) | 4,267 (24.2%) | 105 (30.2%) | 120 (19.6%) |
|  | 2 | 630 (14.6%) | 4,641 (26.3%) | 82 (23.6%) | 213 (34.7%) |
|  | 3 | 2,921 (67.5%) | 5,142 (29.1%) | 114 (32.8%) | 171 (27.9%) |
|  | 4 | 416 (9.6%) | 3,617 (20.5%) | 47 (13.5%) | 109 (17.8%) |
| Wealth Quintile | 1 (lowest) | 364 (8.4%) | 3,960 (22.4%) | 122 (35.1%) | 145 (23.7%) |
|  | 2 | 608 (14.1%) | 3,747 (21.2%) | 83 (23.9%) | 153 (25.0%) |
|  | 3 | 868 (20.1%) | 3,526 (20.0%) | 72 (20.7%) | 126 (20.6%) |
|  | 4 | 1,128 (26.1%) | 3,320 (18.8%) | 41 (11.8%) | 101 (16.5%) |
|  | 5 (highest) | 1,359 (31.4%) | 3,114 (17.6%) | 30 (8.6%) | 88 (14.4%) |
| Type of delivery | Caesarean | 439 (10.1%) | 1,371 (7.8%) | 25 (7.2%) | 40 (6.5%) |
|  | Vaginal | 3,888 (89.9%) | 16,295 (92.2%) | 323 (92.8%) | 573 (93.5%) |
|  | Missing | NA | 1 (0.0%) | NA | NA |
| Maternal Vitamin A Megadose | No | 1,750 (40.4%) | 8,959 (50.7%) | 243 (69.8%) | 339 (55.3%) |
|  | Yes | 2,575 (59.5%) | 8,703 (49.3%) | 104 (29.9%) | 274 (44.7%) |
|  | Missing | 2 (0.0%) | 5 (0.0%) | 1 (0.3%) | NA |

## **Table S7: Ghana descriptive statistics by DTP vaccination**

| Covariate |  | On Time  (42 – 70 days)  n=16,484 | Early  (30 – 42 days)  n=1,096 | Late  (> 70 days)  n=4,524 | Never  n=281 | Missing  n=224 |
| --- | --- | --- | --- | --- | --- | --- |
|  |  | n (%) |  | n (%) | n (%) | n (%) |
| Birthweight | <1.5kg | 46 (0.3%) | 1 (0.1%) | 32 (0.7%) | 3 (1.1%) | NA |
|  | 1.50-1.99kg | 238 (1.4%) | 17 (1.6%) | 121 (2.7%) | 8 (2.8%) | 12 (5.4%) |
|  | 2.00-2.49kg | 2,080 (12.6%) | 136 (12.4%) | 660 (14.6%) | 47 (16.7%) | 38 (17.0%) |
|  | >=2.5kg | 14,120 (85.7%) | 942 (85.9%) | 3,711 (82.0%) | 223 (79.4%) | 174 (77.7%) |
| Head of Household | Father of baby | 12,915 (78.3%) | 853 (77.8%) | 3,566 (78.8%) | 205 (73.0%) | 155 (69.2%) |
|  | Grandfather | 794 (4.8%) | 65 (5.9%) | 246 (5.4%) | 18 (6.4%) | 16 (7.1%) |
|  | Grandmother | 2,238 (13.6%) | 148 (13.5%) | 591 (13.1%) | 45 (16.0%) | 43 (19.2%) |
|  | Mother of baby | 330 (2.0%) | 16 (1.5%) | 72 (1.6%) | 4 (1.4%) | 2 (0.9%) |
|  | Other | 207 (1.3%) | 14 (1.3%) | 49 (1.1%) | 9 (3.2%) | 8 (3.6%) |
| Household Religion | Christian | 11,741 (71.2%) | 746 (68.1%) | 2,967 (65.6%) | 164 (58.4%) | 140 (62.5%) |
|  | Muslim | 3,745 (22.7%) | 271 (24.7%) | 1,238 (27.4%) | 96 (34.2%) | 65 (29.0%) |
|  | None | 769 (4.7%) | 53 (4.8%) | 234 (5.2%) | 15 (5.3%) | 17 (7.6%) |
|  | Trad African | 229 (1.4%) | 26 (2.4%) | 85 (1.9%) | 6 (2.1%) | 2 (0.9%) |
| Maternal age group | <20 | 2,860 (17.4%) | 183 (16.7%) | 920 (20.3%) | 69 (24.6%) | 56 (25.0%) |
|  | 20-24 | 4,302 (26.1%) | 291 (26.6%) | 1,134 (25.1%) | 63 (22.4%) | 75 (33.5%) |
|  | 25-29 | 4,705 (28.5%) | 313 (28.6%) | 1,195 (26.4%) | 76 (27.0%) | 56 (25.0%) |
|  | 30-34 | 2,696 (16.4%) | 182 (16.6%) | 745 (16.5%) | 42 (14.9%) | 25 (11.2%) |
|  | 35-39 | 1,557 (9.4%) | 103 (9.4%) | 422 (9.3%) | 24 (8.5%) | 8 (3.6%) |
|  | 40+ | 364 (2.2%) | 24 (2.2%) | 108 (2.4%) | 7 (2.5%) | 4 (1.8%) |
| Maternal education | None | 4,774 (29.0%) | 368 (33.6%) | 1,672 (37.0%) | 128 (45.6%) | 70 (31.2%) |
|  | Primary | 2,965 (18.0%) | 200 (18.2%) | 904 (20.0%) | 48 (17.1%) | 44 (19.6%) |
|  | Secondary | 8,399 (51.0%) | 513 (46.8%) | 1,911 (42.2%) | 103 (36.7%) | 105 (46.9%) |
|  | Post-Secondary | 346 (2.1%) | 15 (1.4%) | 37 (0.8%) | 2 (0.7%) | 5 (2.2%) |
| Multiple or Singleton Birth | Multiple | 570 (3.5%) | 29 (2.6%) | 188 (4.2%) | 10 (3.6%) | 6 (2.7%) |
|  | Single | 15,914 (96.5%) | 1,067 (97.4%) | 4,336 (95.8%) | 271 (96.4%) | 218 (97.3%) |
| Number of living children in household | 1 | 4,850 (29.4%) | 310 (28.3%) | 1,278 (28.2%) | 79 (28.1%) | 85 (37.9%) |
|  | 2 | 3,759 (22.8%) | 226 (20.6%) | 947 (20.9%) | 60 (21.4%) | 53 (23.7%) |
|  | ≥3 | 7,875 (47.8%) | 560 (51.1%) | 2,299 (50.8%) | 142 (50.5%) | 86 (38.4%) |
| Number of children in household who have died | 0 | 13,404 (81.3%) | 886 (80.8%) | 3,590 (79.4%) | 212 (75.4%) | 188 (83.9%) |
|  | 1 | 2,337 (14.2%) | 149 (13.6%) | 689 (15.2%) | 52 (18.5%) | 29 (12.9%) |
|  | 2 | 563 (3.4%) | 40 (3.6%) | 181 (4.0%) | 12 (4.3%) | 4 (1.8%) |
|  | ≥3 | 180 (1.1%) | 21 (1.9%) | 64 (1.4%) | 5 (1.8%) | 3 (1.3%) |
| Place of birth | Compound | 3,449 (20.9%) | 334 (30.5%) | 1,255 (27.7%) | 94 (33.5%) | 57 (25.4%) |
|  | Facility | 12,979 (78.7%) | 758 (69.2%) | 3,245 (71.7%) | 185 (65.8%) | 167 (74.6%) |
|  | Other | 56 (0.3%) | 4 (0.4%) | 24 (0.5%) | 2 (0.7%) | NA |
| Site ID | 1 | 3,349 (20.3%) | 366 (33.4%) | 934 (20.6%) | 71 (25.3%) | 52 (23.2%) |
|  | 2 | 4,139 (25.1%) | 249 (22.7%) | 936 (20.7%) | 68 (24.2%) | 60 (26.8%) |
|  | 3 | 6,045 (36.7%) | 296 (27.0%) | 1,750 (38.7%) | 80 (28.5%) | 76 (33.9%) |
|  | 4 | 2,951 (17.9%) | 185 (16.9%) | 904 (20.0%) | 62 (22.1%) | 36 (16.1%) |
| Wealth Quintile | 1 (lowest) | 2,962 (18.0%) | 279 (25.5%) | 1,108 (24.5%) | 96 (34.2%) | 50 (22.3%) |
|  | 2 | 3,214 (19.5%) | 236 (21.5%) | 931 (20.6%) | 69 (24.6%) | 51 (22.8%) |
|  | 3 | 3,331 (20.2%) | 215 (19.6%) | 895 (19.8%) | 44 (15.7%) | 44 (19.6%) |
|  | 4 | 3,434 (20.8%) | 183 (16.7%) | 839 (18.5%) | 42 (14.9%) | 38 (17.0%) |
|  | 5 (highest) | 3,543 (21.5%) | 183 (16.7%) | 751 (16.6%) | 30 (10.7%) | 41 (18.3%) |
| Type of delivery | Caesarean | 1,403 (8.5%) | 81 (7.4%) | 341 (7.5%) | 15 (5.3%) | 13 (5.8%) |
|  | Vaginal | 15,080 (91.5%) | 1,015 (92.6%) | 4,183 (92.5%) | 266 (94.7%) | 211 (94.2%) |
|  | Missing | 1 (0.0%) | NA | NA | NA | NA |
| Maternal Vitamin A Megadose | No | 7,782 (47.2%) | 586 (53.5%) | 2,424 (53.6%) | 159 (56.6%) | 123 (54.9%) |
|  | Yes | 8,698 (52.8%) | 509 (46.4%) | 2,098 (46.4%) | 121 (43.1%) | 101 (45.1%) |
|  | Missing | 4 (0.0%) | 1 (0.1%) | 2 (0.0%) | 1 (0.4%) | NA |

## **Table S8: Tanzania descriptive statistics by BCG vaccination**

| Covariate |  | On Time  (≤ 1 day)  n=14,817 | Late  (> 1 day)  n=14,699 | Never  n=783 | Missing  n=1,700 |
| --- | --- | --- | --- | --- | --- |
|  |  | n (%) | n (%) | n (%) | n (%) |
| Birthweight | <1.5kg | 34 (0.2%) | 20 (0.1%) | 2 (0.3%) | 9 (0.5%) |
|  | 1.50-1.99kg | 68 (0.5%) | 111 (0.8%) | 17 (2.2%) | 14 (0.8%) |
|  | 2.00-2.49kg | 2,033 (13.7%) | 1,310 (8.9%) | 78 (10.0%) | 186 (10.9%) |
|  | >=2.5kg | 12,682 (85.6%) | 13,258 (90.2%) | 686 (87.6%) | 1,491 (87.7%) |
| Head of Household | Father of baby | 11,098 (74.9%) | 11,241 (76.5%) | 353 (45.1%) | 1,139 (67.0%) |
|  | Grandfather | 952 (6.4%) | 1,056 (7.2%) | 30 (3.8%) | 129 (7.6%) |
|  | Grandmother | 726 (4.9%) | 676 (4.6%) | 24 (3.1%) | 106 (6.2%) |
|  | Mother of baby | 695 (4.7%) | 860 (5.9%) | 21 (2.7%) | 91 (5.4%) |
|  | Other | 274 (1.8%) | 228 (1.6%) | 8 (1.0%) | 49 (2.9%) |
|  | Missing | 1,072 (7.2%) | 638 (4.3%) | 347 (44.3%) | 186 (10.9%) |
| Household Religion | Christian | 5,097 (34.4%) | 5,998 (40.8%) | 108 (13.8%) | 620 (36.5%) |
|  | Muslim | 3,591 (24.2%) | 4,675 (31.8%) | 76 (9.7%) | 423 (24.9%) |
|  | None | 182 (1.2%) | 452 (3.1%) | 11 (1.4%) | 74 (4.4%) |
|  | Trad African & Other | 29 (0.1%) | 51 (0.3%) | 1 (0.1%) | 9 (0.5%) |
|  | Missing | 5,918 (39.9%) | 3,523 (24.0%) | 587 (75.0%) | 574 (33.8%) |
| Maternal age group | <20 | 2,045 (13.8%) | 1,966 (13.4%) | 81 (10.3%) | 247 (14.5%) |
|  | 20-24 | 4,105 (27.7%) | 4,239 (28.8%) | 151 (19.3%) | 526 (30.9%) |
|  | 25-29 | 4,068 (27.5%) | 3,948 (26.9%) | 119 (15.2%) | 408 (24.0%) |
|  | 30-34 | 2,517 (17.0%) | 2,583 (17.6%) | 76 (9.7%) | 232 (13.6%) |
|  | 35-39 | 1,102 (7.4%) | 1,159 (7.9%) | 24 (3.1%) | 92 (5.4%) |
|  | 40+ | 273 (1.8%) | 268 (1.8%) | 6 (0.8%) | 24 (1.4%) |
|  | Missing | 707 (4.8%) | 536 (3.6%) | 326 (41.6%) | 171 (10.1%) |
| Maternal education | None | 903 (6.1%) | 1,535 (10.4%) | 36 (4.6%) | 182 (10.7%) |
|  | Primary | 11,494 (77.6%) | 11,370 (77.3%) | 350 (44.7%) | 1,214 (71.4%) |
|  | Secondary or more | 1,772 (12.0%) | 1,350 (9.2%) | 73 (9.3%) | 138 (8.1%) |
|  | Missing | 648 (4.4%) | 444 (3.0%) | 324 (41.4%) | 166 (9.8%) |
| Multiple or Singleton Birth | Multiple | 503 (3.4%) | 493 (3.4%) | 38 (4.9%) | 74 (4.4%) |
|  | Single | 14,314 (96.6%) | 14,206 (96.6%) | 745 (95.1%) | 1,626 (95.6%) |
| Number of living children in household | 1 | 3,223 (21.8%) | 3,082 (21.0%) | 149 (19.0%) | 368 (21.6%) |
|  | 2 | 4,334 (29.3%) | 3,986 (27.1%) | 133 (17.0%) | 484 (28.5%) |
|  | ≥3 | 6,574 (44.4%) | 7,146 (48.6%) | 174 (22.2%) | 676 (39.8%) |
|  | Missing | 686 (4.6%) | 485 (3.3%) | 327 (41.8%) | 172 (10.1%) |
| Number of children in household who have died | 0 | 12,057 (81.4%) | 11,838 (80.5%) | 385 (49.2%) | 1,249 (73.5%) |
|  | 1 | 793 (5.4%) | 1,155 (7.9%) | 27 (3.4%) | 128 (7.5%) |
|  | 2 | 368 (2.5%) | 410 (2.8%) | 17 (2.2%) | 43 (2.5%) |
|  | ≥3 | 382 (2.6%) | 392 (2.7%) | 17 (2.2%) | 40 (2.4%) |
|  | Missing | 1,217 (8.2%) | 904 (6.2%) | 337 (43.0%) | 240 (14.1%) |
| Place of birth | Home | 349 (2.4%) | 2,092 (14.2%) | 33 (4.2%) | 179 (10.5%) |
|  | Facility | 13,817 (93.3%) | 11,897 (80.9%) | 632 (80.7%) | 1,430 (84.1%) |
|  | Other | 465 (3.1%) | 574 (3.9%) | 14 (1.8%) | 42 (2.5%) |
|  | Missing | 186 (1.3%) | 136 (0.9%) | 104 (13.3%) | 49 (2.9%) |
| Site ID | Dar es Salaam | 6,765 (45.7%) | 3,921 (26.7%) | 597 (76.2%) | 612 (36.0%) |
|  | Ifakara | 8,052 (54.3%) | 10,778 (73.3%) | 186 (23.8%) | 1,088 (64.0%) |
| Wealth Quintile | 1 (lowest) | 2,476 (16.7%) | 2,970 (20.2%) | 168 (21.5%) | 358 (21.1%) |
|  | 2 | 3,341 (22.5%) | 3,680 (25.0%) | 195 (24.9%) | 451 (26.5%) |
|  | 3 | 1,612 (10.9%) | 1,116 (7.6%) | 166 (21.2%) | 158 (9.3%) |
|  | 4 | 4,636 (31.3%) | 4,611 (31.4%) | 120 (15.3%) | 450 (26.5%) |
|  | 5 (highest) | 2,752 (18.6%) | 2,322 (15.8%) | 134 (17.1%) | 283 (16.6%) |
| Type of delivery | Caesarean | 914 (6.2%) | 833 (5.7%) | 31 (4.0%) | 85 (5.0%) |
|  | Vaginal | 13,903 (93.8%) | 13,866 (94.3%) | 752 (96.0%) | 1,615 (95.0%) |
| Maternal Vitamin A Megadose | No | 3,041 (20.5%) | 6,429 (43.7%) | 251 (32.1%) | 572 (33.6%) |
|  | Yes | 11,569 (78.1%) | 8,088 (55.0%) | 429 (54.8%) | 1,078 (63.4%) |
|  | Missing | 207 (1.4%) | 182 (1.2%) | 103 (13.2%) | 50 (2.9%) |

## **Table S9: Tanzania descriptive statistics by DTP vaccination**

| Covariate |  | On Time  (42 – 70 days)  n=10,851 | Early  (30 – 42 days)  n=15,879 | Late  (> 70 days)  n=1,823 | Never  n=2,257 | Missing  n=96 |
| --- | --- | --- | --- | --- | --- | --- |
|  |  | n (%) | n (%) | n (%) | n (%) | n (%) |
| Birthweight | <1.5kg | 12 (0.1%) | 32 (0.2%) | 6 (0.3%) | 5 (0.2%) | NA |
|  | 1.50-1.99kg | 71 (0.7%) | 76 (0.5%) | 24 (1.3%) | 16 (0.7%) | NA |
|  | 2.00-2.49kg | 953 (8.8%) | 2,134 (13.4%) | 187 (10.3%) | 171 (7.6%) | 8 (8.3%) |
|  | >=2.5kg | 9,815 (90.5%) | 13,637 (85.9%) | 1,606 (88.1%) | 2,065 (91.5%) | 88 (91.7%) |
| Head of Household | Father of baby | 8,339 (76.9%) | 11,972 (75.4%) | 1,361 (74.7%) | 1,534 (68.0%) | 54 (56.2%) |
|  | Grandfather | 750 (6.9%) | 1,064 (6.7%) | 123 (6.7%) | 176 (7.8%) | 4 (4.2%) |
|  | Grandmother | 496 (4.6%) | 787 (5.0%) | 71 (3.9%) | 133 (5.9%) | 7 (7.3%) |
|  | Mother of baby | 606 (5.6%) | 797 (5.0%) | 106 (5.8%) | 122 (5.4%) | 5 (5.2%) |
|  | Other | 166 (1.5%) | 279 (1.8%) | 31 (1.7%) | 62 (2.7%) | 3 (3.1%) |
|  | Missing | 494 (4.6%) | 980 (6.2%) | 131 (7.2%) | 230 (10.2%) | 23 (24.0%) |
| Household Religion | Christian | 4,244 (39.1%) | 6,003 (37.8%) | 555 (30.4%) | 793 (35.1%) | 25 (26.0%) |
|  | Muslim | 3,255 (30.0%) | 4,360 (27.5%) | 442 (24.2%) | 540 (23.9%) | 17 (17.7%) |
|  | None | 248 (2.3%) | 271 (1.7%) | 89 (4.9%) | 93 (4.1%) | 3 (3.1%) |
|  | Trad African & Other | 26 (0.2%) | 41 (0.2%) | 10 (0.5%) | 11 (0.4%) | NA |
|  | Missing | 3,078 (28.4%) | 5,204 (32.8%) | 727 (39.9%) | 820 (36.3%) | 51 (53.1%) |
| Maternal age group | <20 | 1,648 (15.2%) | 1,956 (12.3%) | 263 (14.4%) | 339 (15.0%) | 12 (12.5%) |
|  | 20-24 | 3,102 (28.6%) | 4,465 (28.1%) | 491 (26.9%) | 694 (30.7%) | 22 (22.9%) |
|  | 25-29 | 2,813 (25.9%) | 4,511 (28.4%) | 443 (24.3%) | 563 (24.9%) | 22 (22.9%) |
|  | 30-34 | 1,828 (16.8%) | 2,837 (17.9%) | 310 (17.0%) | 309 (13.7%) | 11 (11.5%) |
|  | 35-39 | 900 (8.3%) | 1,147 (7.2%) | 170 (9.3%) | 105 (4.7%) | 7 (7.3%) |
|  | 40+ | 218 (2.0%) | 260 (1.6%) | 49 (2.7%) | 30 (1.3%) | 2 (2.1%) |
|  | Missing | 342 (3.2%) | 703 (4.4%) | 97 (5.3%) | 217 (9.6%) | 20 (20.8%) |
| Maternal education | None | 959 (8.8%) | 1,195 (7.5%) | 202 (11.1%) | 245 (10.9%) | 4 (4.2%) |
|  | Some Primary | 789 (7.3%) | 1,110 (7.0%) | 185 (10.1%) | 163 (7.2%) | 5 (5.2%) |
|  | Primary | 7,757 (71.5%) | 11,158 (70.3%) | 1,184 (64.9%) | 1,427 (63.2%) | 56 (58.3%) |
|  | Secondary or more | 1,042 (9.6%) | 1,817 (11.4%) | 161 (8.8%) | 214 (9.5%) | 11 (11.5%) |
|  | Missing | 304 (2.8%) | 599 (3.8%) | 91 (5.0%) | 208 (9.2%) | 20 (20.8%) |
| Multiple or Singleton Birth | Multiple | 396 (3.6%) | 472 (3.0%) | 95 (5.2%) | 72 (3.2%) | 4 (4.2%) |
|  | Single | 10,455 (96.4%) | 15,407 (97.0%) | 1,728 (94.8%) | 2,185 (96.8%) | 92 (95.8%) |
| Number of living children in household | 1 | 2,246 (20.7%) | 3,458 (21.8%) | 376 (20.6%) | 499 (22.1%) | 14 (14.6%) |
|  | 2 | 3,131 (28.9%) | 4,516 (28.4%) | 411 (22.5%) | 640 (28.4%) | 30 (31.2%) |
|  | ≥3 | 5,128 (47.3%) | 7,275 (45.8%) | 939 (51.5%) | 903 (40.0%) | 32 (33.3%) |
|  | Missing | 346 (3.2%) | 630 (4.0%) | 97 (5.3%) | 215 (9.5%) | 20 (20.8%) |
| Number of children in household who have died | 0 | 8,446 (77.8%) | 13,245 (83.4%) | 1,459 (80.0%) | 1,710 (75.8%) | 65 (67.7%) |
|  | 1 | 994 (9.2%) | 767 (4.8%) | 131 (7.2%) | 149 (6.6%) | 7 (7.3%) |
|  | 2 | 317 (2.9%) | 393 (2.5%) | 54 (3.0%) | 46 (2.0%) | 1 (1.0%) |
|  | ≥3 | 241 (2.2%) | 460 (2.9%) | 50 (2.7%) | 49 (2.2%) | 3 (3.1%) |
|  | Missing | 853 (7.9%) | 1,014 (6.4%) | 129 (7.1%) | 303 (13.4%) | 20 (20.8%) |
| Place of birth | Home | 1,049 (9.7%) | 1,135 (7.1%) | 225 (12.3%) | 187 (8.3%) | 6 (6.2%) |
|  | Facility | 9,382 (86.5%) | 14,003 (88.2%) | 1,470 (80.6%) | 1,930 (85.5%) | 85 (88.5%) |
|  | Other | 296 (2.7%) | 589 (3.7%) | 98 (5.4%) | 70 (3.1%) | 3 (3.1%) |
|  | Missing | 124 (1.1%) | 152 (1.0%) | 30 (1.6%) | 70 (3.1%) | 2 (2.1%) |
| Site ID | Dar Es Salaam | 3,472 (32.0%) | 5,928 (37.3%) | 832 (45.6%) | 879 (38.9%) | 56 (58.3%) |
|  | Ifakara | 7,379 (68.0%) | 9,951 (62.7%) | 991 (54.4%) | 1,378 (61.1%) | 40 (41.7%) |
| Wealth Quintile | 1 (lowest) | 1,834 (16.9%) | 2,985 (18.8%) | 430 (23.6%) | 472 (20.9%) | 26 (27.1%) |
|  | 2 | 2,654 (24.5%) | 3,607 (22.7%) | 527 (28.9%) | 581 (25.7%) | 21 (21.9%) |
|  | 3 | 927 (8.5%) | 1,492 (9.4%) | 192 (10.5%) | 236 (10.5%) | 19 (19.8%) |
|  | 4 | 3,478 (32.1%) | 5,019 (31.6%) | 477 (26.2%) | 618 (27.4%) | 21 (21.9%) |
|  | 5 (highest) | 1,958 (18.0%) | 2,776 (17.5%) | 197 (10.8%) | 350 (15.5%) | 9 (9.4%) |
| Type of delivery | Caesarean | 611 (5.6%) | 1,030 (6.5%) | 65 (3.6%) | 109 (4.8%) | 2 (2.1%) |
|  | Vaginal | 10,240 (94.4%) | 14,849 (93.5%) | 1,758 (96.4%) | 2,148 (95.2%) | 94 (97.9%) |
| Maternal Vitamin A Megadose | No | 4,025 (37.1%) | 4,402 (27.7%) | 751 (41.2%) | 738 (32.7%) | 35 (36.5%) |
|  | Yes | 6,689 (61.6%) | 11,282 (71.0%) | 1,037 (56.9%) | 1,443 (63.9%) | 59 (61.5%) |
|  | Missing | 137 (1.3%) | 195 (1.2%) | 35 (1.9%) | 76 (3.4%) | 2 (2.1%) |

## **Figure S4: Flow chart of included data from Ghana trial**


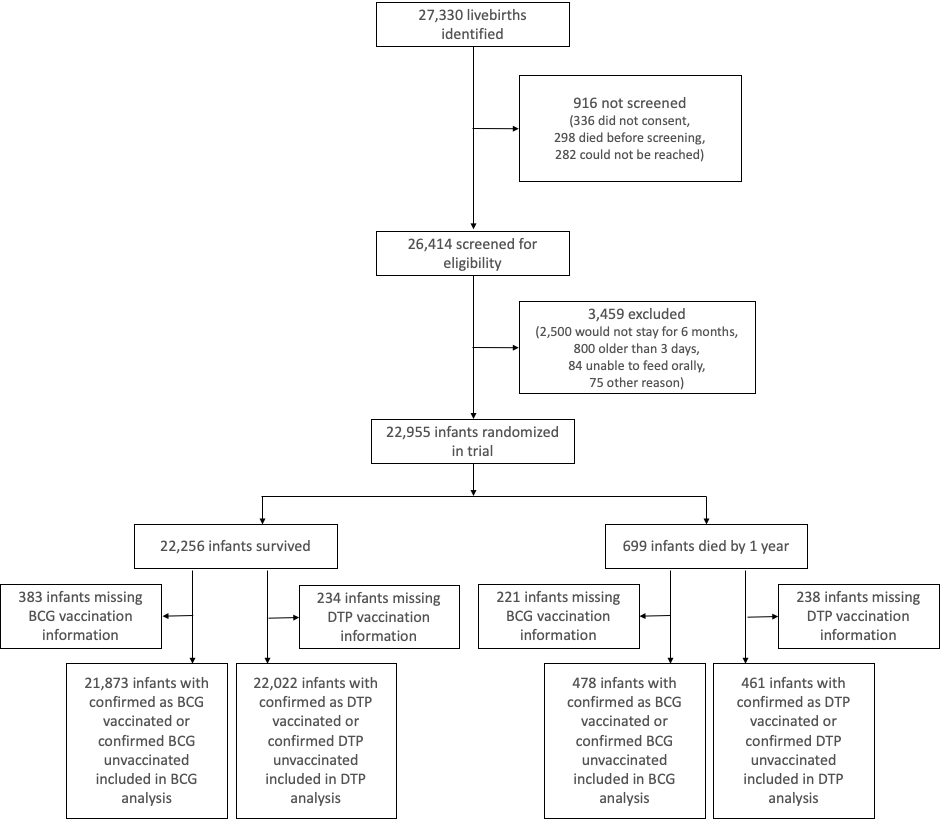


## **Figure S5: Flow chart of included data from Tanzania trial**


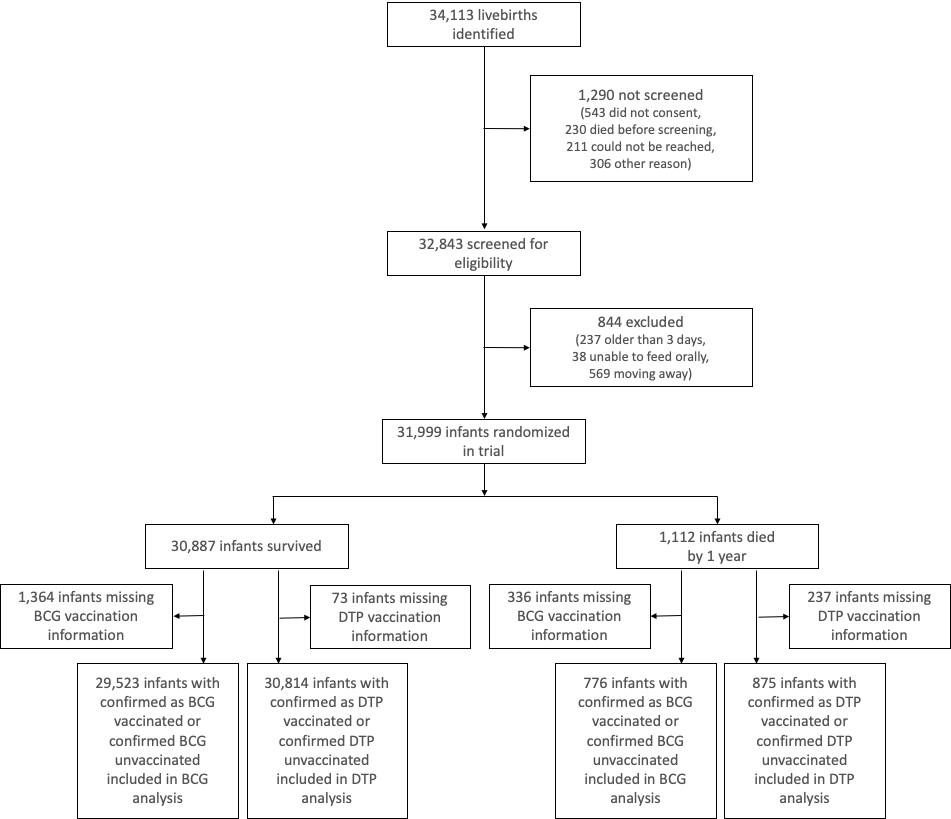


# Bounds Analysis for Vaccination

## **Table S10: Sensitivity Analysis - Missingness Bounds for the association of BCG vaccination with infant mortality^a^**

|  | BCG Vaccinated | | Not BCG Vaccinated | | **Unadjusted^b^**  **HR (95% CI)** | **Adjusted^b^**  **HR (95% CI)** |
| --- | --- | --- | --- | --- | --- | --- |
|  | Number of deaths | Number of infant months | Number of deaths | Number of infant months |  |  |
| **Ghana** |  |  |  |  |  |  |
| Complete Case | 382 | 249,104 | 96 | 16,643 | 0.38 (0.29 - 0.51) | 0.51 (0.38 - 0.68) |
| Vaccinated at baseline | 603 | 253,097 | 96 | 16,643 | 3.69 (2.83 - 4.81) | 4.58 (3.53 - 5.95) |
| Vaccinated at the mode (1 day) | 566 | 253,077 | 133 | 16,663 | 2.03 (1.58 - 2.60) | 2.71 (2.10 - 3.48) |
| Unvaccinated | 391 | 251,912 | 308 | 17,828 | 0.21 (0.17 - 0.26) | 0.28 (0.22 - 0.34) |
| **Tanzania** |  |  |  |  |  |  |
| Complete Case | 573 | 341,332 | 203 | 11,189 | 0.08 (0.07 - 0.10) | 0.08 (0.07 - 0.10) |
| Vaccinated at baseline | 909 | 355707 | 203 | 11189 | 0.65 (0.54 - 0.78) | 0.63 (0.52 - 0.76) |
| Vaccinated at the mode (1 day) | 905 | 355650 | 207 | 11246 | 0.63 (0.52 - 0.75) | 0.61 (0.51 - 0.73) |
| Unvaccinated | 641 | 354637 | 471 | 12259 | 0.08 (0.07 - 0.1) | 0.08 (0.06 - 0.09) |

^a^ This sensitivity analysis corresponds to Table 1 in the main paper.

^b^ All hazard ratios are calculated using Cox proportional hazards models allowing for time-varying vaccination status and are controlled for continuous birthweight (with spline knots at 1.5, 2, 2.5, and 3.5kg), head of household (mother, father, grandmother, grandfather, other), household religion (Christian, Muslim, None, Traditional African), maternal age (<20, 20-24, 25-29, 30-34, 35-39, ≥40), maternal education (None, Primary, Secondary for Tanzania, and additionally Post-secondary for Ghana), multiple or singleton birth, number of living children in household (0, 1, 2, 3+), number of children in household who have died (0, 1, 2, 3+), place of birth (Home, Facility, Other), site ID (1-4 for Ghana and 1-2 for Tanzania), wealth quintile, delivery type (vaginal or caesarean), and maternal megadose of vitamin A.

## **Table S11: Sensitivity Analysis - Missingness Bounds for the association of DTP vaccination with infant mortality, from 30 to 365 days^a^**

|  | DTP Vaccinated | | Not DTP Vaccinated | | **Unadjusted^b^**  **HR (95% CI)** | **Adjusted^b^**  **HR (95% CI)** |
| --- | --- | --- | --- | --- | --- | --- |
|  | Number of deaths | Number of infant months | Number of deaths | Number of infant months |  |  |
| **Ghana** |  |  |  |  |  |  |
| Complete Case | 298 | 219,008 | 69 | 24,860 | 0.34 (0.23 - 0.52) | 0.39 (0.26 - 0.59) |
| Vaccinated at baseline | 336 | 220,891 | 69 | 24,860 | 1.38 (0.87 - 2.19) | 1.56 (0.99 - 2.47) |
| Vaccinated at the mode (45 days) | 316 | 220,785 | 89 | 24,962 | 0.51 (0.34 - 0.78) | 0.59 (0.39 - 0.89) |
| Unvaccinated | 298 | 219,280 | 107 | 26,472 | 0.31 (0.22 - 0.45) | 0.36 (0.25 - 0.51) |
| **Tanzania** |  |  |  |  |  |  |
| Complete Case | 402 | 282,709 | 234 | 34,066 | 0.19 (0.16 - 0.22) | 0.19 (0.16 - 0.23) |
| Vaccinated at baseline | 425 | 283491 | 234 | 34066 | 0.2 (0.17 - 0.24) | 0.2 (0.17 - 0.24) |
| Vaccinated at the mode (31 days) | 425 | 283488 | 234 | 34069 | 0.2 (0.17 - 0.24) | 0.2 (0.17 - 0.24) |
| Unvaccinated | 422 | 283428 | 237 | 34129 | 0.2 (0.17 - 0.24) | 0.2 (0.17 - 0.24) |

^a^ This sensitivity analysis corresponds to Table 2 in the main paper.

^b^ All hazard ratios are calculated using Cox proportional hazards models allowing for time-varying vaccination status and are controlled for continuous birthweight (with spline knots at 1.5, 2, 2.5, and 3.5kg), head of household (mother, father, grandmother, grandfather, other), household religion (Christian, Muslim, None, Traditional African), maternal age (<20, 20-24, 25-29, 30-34, 35-39, ≥40), maternal education (None, Primary, Secondary for Tanzania, and additionally Post-secondary for Ghana), multiple or singleton birth, number of living children in household (0, 1, 2, 3+), number of children in household who have died (0, 1, 2, 3+), place of birth (Home, Facility, Other), site ID (1-4 for Ghana and 1-2 for Tanzania), wealth quintile, delivery type (vaginal or caesarean), and maternal megadose of vitamin A.

# Combined Estimates

## **Table S12: Combined country estimates for the adjusted effect of Vitamin A Supplementation on Infant Mortality, stratified by BCG Vaccination Status and Infant Sex^a^**

|  |  | **Combined, Adjusted Effect of Vitamin A on Infant Mortality Stratified by BCG Status^b^**  **HR (95% CI)** |
| --- | --- | --- |
|  |  |  |
| **All**  **Infants** | Unvaccinated Time | 1.29 (1.03 - 1.63) |
|  | Vaccinated Time | 1.05 (0.92 - 1.19) |
| **Female Infants** | Unvaccinated Time | 1.42 (1.00 – 2.04) |
|  | Vaccinated Time | 1.06 (0.88 - 1.27) |
| **Male**  **Infants** | Unvaccinated Time | 1.18 (0.87 – 1.59) |
|  | Vaccinated Time | 1.04 (0.87 – 1.24) |

^a^ These estimates correspond with estimates presented in Table 3 of the main paper. See figures S1a and S1b for additional details.

^b^ Hazard Ratios were combined by through a fixed-effect metanalysis of the adjusted estimates from Tanzania and Ghana. All hazard ratios are calculated using Cox proportional hazards models allowing for time-varying vaccination status and are controlled for continuous birthweight (with spline knots at 1.5, 2, 2.5, and 3.5kg), head of household (mother, father, grandmother, grandfather, other), household religion (Christian, Muslim, None, Traditional African), maternal age (<20, 20-24, 25-29, 30-34, 35-39, ≥40), maternal education (None, Primary, Secondary for Tanzania, and additionally Post-secondary for Ghana), multiple or singleton birth, number of living children in household (0, 1, 2, 3+), number of children in household who have died (0, 1, 2, 3+), place of birth (Home, Facility, Other), site ID (1-4 for Ghana and 1-2 for Tanzania), wealth quintile, delivery type (vaginal or caesarean), and maternal megadose of vitamin A.

## **Figure S6a: Combined forest plot of the adjusted effect of vitamin A supplementation on infant mortality, stratified by BCG vaccination status^a^**





^a^ This figure is a graphical representation of the estimates presented in supplemental table S8.

## **Figure S6b: Combined forest plot of the adjusted effect of vitamin A supplementation on infant mortality, stratified by BCG vaccination status and sex^a^**





^a^ This figure is a graphical representation of the estimates presented in supplemental table S8 by sex.

## **Table S13: Combined country estimates for the adjusted effect of vitamin A supplementation on infant mortality, stratified by DTP vaccination status and infant sex^a^**

|  |  | **Combined, Adjusted Effect of Vitamin A on Infant Mortality Stratified by DTP Status^b^**  **HR (95% CI)** |
| --- | --- | --- |
|  |  |  |
| **All**  **Infants** | Unvaccinated Time | 1.05 (0.83 - 1.31) |
|  | Vaccinated Time | 1.14 (0.98 - 1.32) |
| **Female Infants** | Unvaccinated Time | 1.07 (0.75 – 1.51) |
|  | Vaccinated Time | 1.22 (0.99 - 1.51) |
| **Male**  **Infants** | Unvaccinated Time | 1.03 (0.76 – 1.39) |
|  | Vaccinated Time | 1.07 (0.87 – 1.32) |

^a^ These estimates correspond with estimates presented in Table 3 of the main paper. See figures S2a and S2b for additional details.

^b^ Hazard Ratios were combined by through a fixed-effect metanalysis of the adjusted estimates from Tanzania and Ghana. All hazard ratios are calculated using Cox proportional hazards models allowing for time-varying vaccination status and are controlled for continuous birthweight (with spline knots at 1.5, 2, 2.5, and 3.5kg), head of household (mother, father, grandmother, grandfather, other), household religion (Christian, Muslim, None, Traditional African), maternal age (<20, 20-24, 25-29, 30-34, 35-39, ≥40), maternal education (None, Primary, Secondary for Tanzania, and additionally Post-secondary for Ghana), multiple or singleton birth, number of living children in household (0, 1, 2, 3+), number of children in household who have died (0, 1, 2, 3+), place of birth (Home, Facility, Other), site ID (1-4 for Ghana and 1-2 for Tanzania), wealth quintile, delivery type (vaginal or caesarean), and maternal megadose of vitamin A.

# Bounds Analysis for Neonatal Vitamin A – Vaccine Interaction

## **Table S14: Sensitivity Analysis - Bounds of the adjusted effect of vitamin A supplementation on infant mortality, stratified by BCG vaccination status for all infants^a^**

|  |  | **Vitamin A** | | **Placebo** | | **Vitamin A Hazard Ratio**  **for Mortality^b^**  **HR (95% CI)** | **P-value for**  **Vitamin A and BCG Interaction** |
| --- | --- | --- | --- | --- | --- | --- | --- |
|  |  | Number of deaths | Number of infant months | Number of deaths | Number of infant months |  |  |
| **Ghana** |  |  |  |  |  |  |  |
| **Complete Case** | Unvaccinated Time | 53 | 8,264 | 43 | 8,378 | 1.26 (0.84 - 1.89) | 0.44 |
|  | Vaccinated Time | 195 | 124,193 | 187 | 124,911 | 1.06 (0.86 - 1.29) |  |
| **Vaccinated at baseline** | Unvaccinated Time | 53 | 8,264 | 43 | 8,378 | 1.26 (0.84 - 1.89) | 0.61 |
|  | Vaccinated Time | 318 | 126,310 | 285 | 126,788 | 1.13 (0.96 - 1.32) |  |
| **Vaccinated at mode (1 day)** | Unvaccinated Time | 74 | 8,275 | 59 | 8,388 | 1.27 (0.90 - 1.80) | 0.51 |
|  | Vaccinated Time | 297 | 126,299 | 269 | 126,778 | 1.12 (0.95 - 1.32) |  |
| **Unvaccinated** | Unvaccinated Time | 172 | 8,837 | 136 | 8,991 | 1.28 (1.02 - 1.60) | 0.21 |
|  | Vaccinated Time | 199 | 125,737 | 192 | 126,175 | 1.05 (0.86 - 1.28) |  |
| **Tanzania** |  |  |  |  |  |  |  |
| **Complete Case** | Unvaccinated Time | 115 | 5,661 | 88 | 5,528 | 1.31 (0.99 - 1.73) | 0.17 |
|  | Vaccinated Time | 291 | 170,382 | 282 | 170,950 | 1.04 (0.88 - 1.23) |  |
| **Vaccinated at baseline** | Unvaccinated Time | 115 | 5,661 | 88 | 5,528 | 1.33 (1.01 - 1.75) | 0.07 |
|  | Vaccinated Time | 451 | 177,489 | 458 | 178,218 | 1.00 (0.87 - 1.13) |  |
| **Vaccinated at mode (1 day)** | Unvaccinated Time | 117 | 5689 | 90 | 5557 | 1.32 (1 - 1.74) | 0.07 |
|  | Vaccinated Time | 449 | 177461 | 456 | 178189 | 1 (0.87 - 1.14) |  |
| **Unvaccinated** | Unvaccinated Time | 243 | 6191 | 228 | 6068 | 1.08 (0.9 - 1.29) | 0.69 |
|  | Vaccinated Time | 323 | 176959 | 318 | 177678 | 1.03 (0.88 - 1.2) |  |

^a^ This sensitivity analysis corresponds to Table 3 in the main paper.

^b^ All hazard ratios are calculated using Cox proportional hazards models allowing for time-varying vaccination status and are controlled for continuous birthweight (with spline knots at 1.5, 2, 2.5, and 3.5kg), head of household (mother, father, grandmother, grandfather, other), household religion (Christian, Muslim, None, Traditional African), maternal age (<20, 20-24, 25-29, 30-34, 35-39, ≥40), maternal education (None, Primary, Secondary for Tanzania, and additionally Post-secondary for Ghana), multiple or singleton birth, number of living children in household (0, 1, 2, 3+), number of children in household who have died (0, 1, 2, 3+), place of birth (Home, Facility, Other), site ID (1-4 for Ghana and 1-2 for Tanzania), wealth quintile, delivery type (vaginal or caesarean), and maternal megadose of vitamin A.

## **Table S15: Sensitivity Analysis - Bounds of the adjusted effect of vitamin A supplementation on infant mortality, stratified by BCG vaccination status for female infants^a^**

|  |  | **Vitamin A** | | **Placebo** | | **Vitamin A Hazard Ratio for Mortality^b^**  **HR (95% CI)** | **P-value for**  **Vitamin A and BCG Interaction** |
| --- | --- | --- | --- | --- | --- | --- | --- |
|  |  | Number of deaths | Number of infant months | Number of deaths | Number of infant months |  |  |
| **Ghana** |  |  |  |  |  |  |  |
| **Complete Case** | Unvaccinated Time | 25 | 3,963 | 19 | 4,096 | 1.28 (0.70 - 2.34) | 0.35 |
|  | Vaccinated Time | 90 | 60,993 | 99 | 62,077 | 0.93 (0.70 - 1.24) |  |
| **Vaccinated at baseline** | Unvaccinated Time | 25 | 3,963 | 19 | 4,096 | 1.31 (0.72 - 2.38) | 0.54 |
|  | Vaccinated Time | 147 | 61,892 | 143 | 63,040 | 1.07 (0.85 - 1.35) |  |
| **Vaccinated at mode (1 day)** | Unvaccinated Time | 36 | 3,968 | 25 | 4,100 | 1.44 (0.86 - 2.40) | 0.25 |
|  | Vaccinated Time | 136 | 61,888 | 137 | 63,036 | 1.03 (0.81 - 1.31) |  |
| **Unvaccinated** | Unvaccinated Time | 81 | 4,219 | 62 | 4,367 | 1.32 (0.95 - 1.84) | 0.12 |
|  | Vaccinated Time | 91 | 61,636 | 100 | 62,768 | 0.94 (0.71 - 1.25) |  |
| **Tanzania** |  |  |  |  |  |  |  |
| **Complete Case** | Unvaccinated Time | 51 | 2,714 | 32 | 2,634 | 1.51 (0.97 - 2.36) | 0.30 |
|  | Vaccinated Time | 136 | 80,636 | 121 | 81,959 | 1.16 (0.91 - 1.48) |  |
| **Vaccinated at baseline** | Unvaccinated Time | 51 | 2,714 | 32 | 2,634 | 1.62 (1.04 - 2.52) | 0.11 |
|  | Vaccinated Time | 203 | 84,034 | 190 | 85,502 | 1.10 (0.90 - 1.34) |  |
| **Vaccinated at mode (1 day)** | Unvaccinated Time | 52 | 2727 | 32 | 2648 | 1.65 (1.06 - 2.56) | 0.10 |
|  | Vaccinated Time | 202 | 84021 | 190 | 85489 | 1.1 (0.9 - 1.34) |  |
| **Unvaccinated** | Unvaccinated Time | 106 | 2965 | 87 | 2941 | 1.22 (0.92 - 1.62) | 0.68 |
|  | Vaccinated Time | 148 | 83782 | 135 | 85195 | 1.13 (0.89 - 1.43) |  |

^a^ This sensitivity analysis corresponds to female infant estimates from Table 3 in the main paper.

^b^  All hazard ratios are calculated using Cox proportional hazards models allowing for time-varying vaccination status and are controlled for continuous birthweight (with spline knots at 1.5, 2, 2.5, and 3.5kg), head of household (mother, father, grandmother, grandfather, other), household religion (Christian, Muslim, None, Trad African), maternal age (<20, 20-24, 25-29, 30-34, 35-39, ≥40), maternal education (None, Primary, Secondary for Tanzania, and additionally Post-secondary for Tanzania), multiple or singleton birth, number of living children in household (0, 1, 2, 3+), number of children in household who have died (0, 1, 2, 3+), place of birth (Compound, Facility, Other), site ID (1-4 for Ghana and 1-2 for Tanzania), wealth quintile, delivery type (vaginal or caesarean), and maternal megadose of vitamin A.

## **Table S16: Sensitivity Analysis - Bounds of the adjusted effect of vitamin A supplementation on infant mortality, stratified by BCG vaccination status for male infants^a^**

|  |  | **Vitamin A** | | **Placebo** | | **Vitamin A Hazard Ratio for Mortality^b^**  **HR (95% CI)** | **P-value for**  **Vitamin A and BCG Interaction** |
| --- | --- | --- | --- | --- | --- | --- | --- |
|  |  | Number of deaths | Number of infant months | Number of deaths | Number of infant months |  |  |
| **Ghana: Male Infants** | |  |  |  |  |  |  |
| **Complete Case** | Unvaccinated Time | 28 | 4,302 | 24 | 4,283 | 1.24 (0.71 - 2.15) | 0.94 |
|  | Vaccinated Time | 105 | 63,200 | 88 | 62,834 | 1.21 (0.91 - 1.60) |  |
| **Vaccinated at baseline** | Unvaccinated Time | 28 | 4,302 | 24 | 4,283 | 1.22 (0.70 - 2.11) | 0.96 |
|  | Vaccinated Time | 171 | 64,417 | 142 | 63,748 | 1.20 (0.96 - 1.50) |  |
| **Vaccinated at mode (1 day)** | Unvaccinated Time | 38 | 4,308 | 34 | 4,287 | 1.14 (0.71 - 1.82) | 0.79 |
|  | Vaccinated Time | 161 | 64,411 | 132 | 63,743 | 1.22 (0.97 - 1.54) |  |
| **Unvaccinated** | Unvaccinated Time | 91 | 4,618 | 74 | 4,624 | 1.24 (0.91 - 1.69) | 0.85 |
|  | Vaccinated Time | 108 | 64,100 | 92 | 63,407 | 1.19 (0.90 - 1.57) |  |
| **Tanzania: Male Infants** | |  |  |  |  |  |  |
| **Complete Case** | Unvaccinated Time | 64 | 2,947 | 56 | 2,894 | 1.16 (0.81 - 1.66) | 0.37 |
|  | Vaccinated Time | 155 | 89,722 | 161 | 88,979 | 0.95 (0.76 - 1.19) |  |
| **Vaccinated at baseline** | Unvaccinated Time | 64 | 2,947 | 56 | 2,894 | 1.14 (0.79 - 1.63) | 0.30 |
|  | Vaccinated Time | 248 | 93,455 | 268 | 92,716 | 0.92 (0.77 - 1.09) |  |
| **Vaccinated at mode (1 day)** | Unvaccinated Time | 65 | 2962 | 58 | 2909 | 1.11 (0.78 - 1.59) | 0.35 |
|  | Vaccinated Time | 247 | 93440 | 266 | 92701 | 0.92 (0.78 - 1.1) |  |
| **Unvaccinated** | Unvaccinated Time | 137 | 3225 | 141 | 3127 | 0.96 (0.76 - 1.22) | 0.93 |
|  | Vaccinated Time | 175 | 93176 | 183 | 92483 | 0.95 (0.77 - 1.17) |  |

^a^ This sensitivity analysis corresponds to male infant estimates from Table 3 in the main paper.

^b^ All hazard ratios are calculated using Cox proportional hazards models allowing for time-varying vaccination status and are controlled for continuous birthweight (with spline knots at 1.5, 2, 2.5, and 3.5kg), head of household (mother, father, grandmother, grandfather, other), household religion (Christian, Muslim, None, Trad African), maternal age (<20, 20-24, 25-29, 30-34, 35-39, ≥40), maternal education (None, Primary, Secondary for Tanzania, and additionally Post-secondary for Tanzania), multiple or singleton birth, number of living children in household (0, 1, 2, 3+), number of children in household who have died (0, 1, 2, 3+), place of birth (Compound, Facility, Other), site ID (1-4 for Ghana and 1-2 for Tanzania), wealth quintile, delivery type (vaginal or caesarean), and maternal megadose of vitamin A.

## **Table S17: Sensitivity Analysis - Bounds of the adjusted effect of vitamin A supplementation on infant mortality, stratified by DTP vaccination status for all infants^a^**

|  |  | **Vitamin A** | | **Placebo** | | **Vitamin A Hazard Ratio for Mortality^b^**  **HR (95% CI)** | **P-value for**  **Vitamin A and DTP Interaction** |
| --- | --- | --- | --- | --- | --- | --- | --- |
|  |  | Number of deaths | Number of infant months | Number of deaths | Number of infant months |  |  |
| **Ghana** |  |  |  |  |  |  |  |
| **Complete Case** | Unvaccinated Time | 31 | 12,327 | 38 | 12,533 | 0.87 (0.54 - 1.41) | 0.18 |
|  | Vaccinated Time | 165 | 109,301 | 133 | 109,707 | 1.26 (1.00 - 1.58) |  |
| **Vaccinated at baseline** | Unvaccinated Time | 31 | 12,327 | 38 | 12,533 | 0.85 (0.53 - 1.37) | 0.15 |
|  | Vaccinated Time | 186 | 110,312 | 150 | 110,580 | 1.25 (1.00 - 1.55) |  |
| **Vaccinated at mode**  **(45 days)** | Unvaccinated Time | 43 | 12,382 | 46 | 12,584 | 0.98 (0.64 - 1.48) | 0.33 |
|  | Vaccinated Time | 174 | 110,257 | 142 | 110,528 | 1.23 (0.99 - 1.54) |  |
| **Unvaccinated** | Unvaccinated Time | 52 | 13,166 | 55 | 13,306 | 0.98 (0.67 - 1.43) | 0.28 |
|  | Vaccinated Time | 165 | 109,473 | 133 | 109,806 | 1.25 (0.99 - 1.57) |  |
| **Tanzania** |  |  |  |  |  |  |  |
| **Complete Case** | Unvaccinated Time | 123 | 17,100 | 111 | 16,966 | 1.10 (0.85 - 1.42) | 0.80 |
|  | Vaccinated Time | 205 | 141,196 | 197 | 141,513 | 1.05 (0.87 - 1.28) |  |
| **Vaccinated at baseline** | Unvaccinated Time | 123 | 17100 | 111 | 16966 | 1.10 (0.85 - 1.42) | 0.68 |
|  | Vaccinated Time | 214 | 141510 | 211 | 141981 | 1.03 (0.85 - 1.24) |  |
| **Vaccinated at mode**  **(31 days)** | Unvaccinated Time | 123 | 17101 | 111 | 16968 | 1.1 (0.85 - 1.42) | 0.68 |
|  | Vaccinated Time | 214 | 141509 | 211 | 141979 | 1.03 (0.85 - 1.24) |  |
| **Unvaccinated** | Unvaccinated Time | 124 | 17124 | 113 | 17005 | 1.09 (0.84 - 1.41) | 0.74 |
|  | Vaccinated Time | 213 | 141486 | 209 | 141942 | 1.03 (0.85 - 1.25) |  |

^a^ This sensitivity analysis corresponds to estimates from Table 4 in the main paper.

^b^ All hazard ratios are calculated using Cox proportional hazards models allowing for time-varying vaccination status and are controlled for continuous birthweight (with spline knots at 1.5, 2, 2.5, and 3.5kg), head of household (mother, father, grandmother, grandfather, other), household religion (Christian, Muslim, None, Trad African), maternal age (<20, 20-24, 25-29, 30-34, 35-39, ≥40), maternal education (None, Primary, Secondary for Tanzania, and additionally Post-secondary for Tanzania), multiple or singleton birth, number of living children in household (0, 1, 2, 3+), number of children in household who have died (0, 1, 2, 3+), place of birth (Compound, Facility, Other), site ID (1-4 for Ghana and 1-2 for Tanzania), wealth quintile, delivery type (vaginal or caesarean), and maternal megadose of vitamin A.

## **Table S18: Sensitivity Analysis - Bounds of the adjusted effect of vitamin A supplementation on infant mortality, stratified by DTP vaccination status for female infants^a^**

|  |  | **Vitamin A** | | **Placebo** | | **Vitamin A Hazard Ratio for Mortality^b^**  **HR (95% CI)** | **P-value for**  **Vitamin A and DTP Interaction** |
| --- | --- | --- | --- | --- | --- | --- | --- |
|  |  | Number of deaths | Number of infant months | Number of deaths | Number of infant months |  |  |
| **Ghana: Female Infants** | |  |  |  |  |  |  |
| **Complete Case** | Unvaccinated Time | 13 | 5,898 | 19 | 6,224 | 0.75 (0.37 - 1.52) | 0.26 |
|  | Vaccinated Time | 79 | 53,589 | 70 | 54,526 | 1.16 (0.84 - 1.60) |  |
| **Vaccinated at baseline** | Unvaccinated Time | 13 | 5,898 | 19 | 6,224 | 0.72 (0.36 - 1.46) | 0.20 |
|  | Vaccinated Time | 87 | 54,089 | 75 | 54,916 | 1.19 (0.87 - 1.62) |  |
| **Vaccinated at mode**  **(45 days)** | Unvaccinated Time | 20 | 5,923 | 23 | 6,246 | 0.93 (0.51 - 1.70) | 0.54 |
|  | Vaccinated Time | 80 | 54,063 | 71 | 54,894 | 1.16 (0.84 - 1.59) |  |
| **Unvaccinated** | Unvaccinated Time | 21 | 6,312 | 24 | 6,561 | 0.93 (0.52 - 1.68) | 0.52 |
|  | Vaccinated Time | 79 | 53,674 | 70 | 54,579 | 1.16 (0.84 - 1.60) |  |
| **Tanzania: Female Infants** | |  |  |  |  |  |  |
| **Complete Case** | Unvaccinated Time | 54 | 8,157 | 45 | 8,113 | 1.19 (0.80 - 1.77) | 0.78 |
|  | Vaccinated Time | 103 | 66,783 | 83 | 67,665 | 1.27 (0.95 - 1.70) |  |
| **Vaccinated at baseline** | Unvaccinated Time | 54 | 8157 | 45 | 8113 | 1.19 (0.8 - 1.77) | 0.88 |
|  | Vaccinated Time | 107 | 66951 | 89 | 67946 | 1.23 (0.93 - 1.64) |  |
| **Vaccinated at mode**  **(31 days)** | Unvaccinated Time | 54 | 8157 | 45 | 8114 | 1.19 (0.8 - 1.77) | 0.88 |
|  | Vaccinated Time | 107 | 66950 | 89 | 67945 | 1.23 (0.93 - 1.64) |  |
| **Unvaccinated** | Unvaccinated Time | 55 | 8169 | 45 | 8136 | 1.21 (0.82 - 1.8) | 0.98 |
|  | Vaccinated Time | 106 | 66938 | 89 | 67923 | 1.22 (0.92 - 1.62) |  |

^a^ This sensitivity analysis corresponds to the female estimates from Table 4 in the main paper.

^b^ All hazard ratios are calculated using Cox proportional hazards models allowing for time-varying vaccination status and are controlled for continuous birthweight (with spline knots at 1.5, 2, 2.5, and 3.5kg), head of household (mother, father, grandmother, grandfather, other), household religion (Christian, Muslim, None, Trad African), maternal age (<20, 20-24, 25-29, 30-34, 35-39, ≥40), maternal education (None, Primary, Secondary for Tanzania, and additionally Post-secondary for Tanzania), multiple or singleton birth, number of living children in household (0, 1, 2, 3+), number of children in household who have died (0, 1, 2, 3+), place of birth (Compound, Facility, Other), site ID (1-4 for Ghana and 1-2 for Tanzania), wealth quintile, delivery type (vaginal or caesarean), and maternal megadose of vitamin A.

## **Table S19: Sensitivity Analysis - Bounds of the adjusted effect of vitamin A supplementation on infant mortality, stratified by DTP vaccination status for male infants^a^**

|  |  | **Vitamin A** | | **Placebo** | | **Vitamin A Hazard Ratio for Mortality^b^**  **HR (95% CI)** | **P-value for**  **Vitamin A and DTP Interaction** |
| --- | --- | --- | --- | --- | --- | --- | --- |
|  |  | Number of deaths | Number of infant months | Number of deaths | Number of infant months |  |  |
| **Ghana: Male Infants** | |  |  |  |  |  |  |
| **Complete Case** | Unvaccinated Time | 18 | 6,430 | 19 | 6,309 | 1.00 (0.52 - 1.92) | 0.39 |
|  | Vaccinated Time | 86 | 55,712 | 63 | 55,181 | 1.38 (0.99 - 1.91) |  |
| **Vaccinated at baseline** | Unvaccinated Time | 18 | 6,430 | 19 | 6,309 | 0.99 (0.52 - 1.91) | 0.43 |
|  | Vaccinated Time | 99 | 56,223 | 75 | 55,664 | 1.32 (0.98 - 1.78) |  |
| **Vaccinated at mode**  **(45 days)** | Unvaccinated Time | 23 | 6,459 | 23 | 6,339 | 1.03 (0.57 - 1.85) | 0.54 |
|  | Vaccinated Time | 94 | 56,194 | 71 | 55,634 | 1.33 (0.97 - 1.81) |  |
| **Unvaccinated** | Unvaccinated Time | 31 | 6,854 | 31 | 6,745 | 1.02 (0.61 - 1.68) | 0.33 |
|  | Vaccinated Time | 86 | 55,799 | 63 | 55,227 | 1.37 (0.99 - 1.90) |  |
| **Tanzania: Male Infants** | |  |  |  |  |  |  |
| **Complete Case** | Unvaccinated Time | 69 | 8,943 | 66 | 8,840 | 1.04 (0.74 - 1.46) | 0.50 |
|  | Vaccinated Time | 102 | 74,391 | 114 | 73,838 | 0.89 (0.68 - 1.17) |  |
| **Vaccinated at baseline** | Unvaccinated Time | 69 | 8943 | 66 | 8853 | 1.04 (0.74 - 1.45) | 0.44 |
|  | Vaccinated Time | 107 | 74560 | 122 | 74034 | 0.88 (0.67 - 1.13) |  |
| **Vaccinated at mode**  **(31 days)** | Unvaccinated Time | 69 | 8944 | 66 | 8854 | 1.04 (0.74 - 1.45) | 0.44 |
|  | Vaccinated Time | 107 | 74559 | 122 | 74033 | 0.88 (0.67 - 1.13) |  |
| **Unvaccinated** | Unvaccinated Time | 69 | 8955 | 68 | 8868 | 1.01 (0.72 - 1.41) | 0.57 |
|  | Vaccinated Time | 107 | 74548 | 120 | 74019 | 0.89 (0.69 - 1.16) |  |

^a^ This sensitivity analysis corresponds to the male estimates from Table 4 in the main paper.

^b^ All hazard ratios are calculated using Cox proportional hazards models allowing for time-varying vaccination status and are controlled for continuous birthweight (with spline knots at 1.5, 2, 2.5, and 3.5kg), head of household (mother, father, grandmother, grandfather, other), household religion (Christian, Muslim, None, Trad African), maternal age (<20, 20-24, 25-29, 30-34, 35-39, ≥40), maternal education (None, Primary, Secondary for Tanzania, and additionally Post-secondary for Tanzania), multiple or singleton birth, number of living children in household (0, 1, 2, 3+), number of children in household who have died (0, 1, 2, 3+), place of birth (Compound, Facility, Other), site ID (1-4 for Ghana and 1-2 for Tanzania), wealth quintile, delivery type (vaginal or caesarean), and maternal megadose of vitamin A.

# Effect modification by previous BCG

## **Table S20: Effect modification by previous BCG on the association of DTP and survival**

|  | | DTP Vaccinated | | Not DTP Vaccinated | | **DTP Unadjusted**  **HR (95% CI)**  **for Mortality^a^** | **DTP Adjusted HR**  **(95% CI) for Mortality^b^** |  |
| --- | --- | --- | --- | --- | --- | --- | --- | --- |
|  | | Number of deaths | Number of infant months at risk | Number of deaths | Number of infant months at risk |  |  | p-value for interaction for DTP and prior BCG |
| **Ghana** | |  |  |  |  |  |  |  |
| Full population | | 298 | 219,008 | 69 | 24,860 | 0.34 (0.23, 0.52) | 0.39 (0.26, 0.59) | - |
| Population by BCG status | BCG Vaccinated Infants | 273 | 207,899 | 48 | 22,872 | 0.39 (0.24 - 0.62) | 0.43 (0.27 - 0.69) | < 0.001 |
|  | BCG Unvaccinated Infants | 17 | 8,821 | 21 | 1,508 | 0.22 (0.09 - 0.53) | 0.17 (0.06 - 0.48) |  |
| **Tanzania** |  |  |  |  |  |  |  |  |
| Full population | | 402 | 282,709 | 234 | 34,066 | 0.19 (0.16, 0.22) | 0.19 (0.16, 0.22) | - |
| Population by BCG status | BCG Vaccinated Infants | 385 | 271,930 | 107 | 21,602 | 0.19 (0.15 - 0.25) | 0.20 (0.15 - 0.25) | < 0.001 |
|  | BCG Unvaccinated Infants | 11 | 9,253 | 75 | 1,558 | 0.03 (0.01 - 0.05) | 0.04 (0.02 - 0.07) |  |

^a^ All analyses were conducted using Cox proportional hazard models allowing for time-varying vaccination status.

^b^ Adjusted models controlled for continuous birthweight (with spline knots at 1.5, 2, 2.5, and 3.5kg), head of household (mother, father, grandmother, grandfather, other), household religion (Christian, Muslim, None, Traditional African), maternal age (<20, 20-24, 25-29, 30-34, 35-39, ≥40), maternal education (None, Primary, Secondary for Tanzania, and additionally Post-secondary for Ghana), multiple or singleton birth, number of living children in household (0, 1, 2, 3+), number of children in household who have died (0, 1, 2, 3+), place of birth (Home, Facility, Other), site ID (1-4 for Ghana and 1-2 for Tanzania), wealth quintile, delivery type (vaginal or caesarean), and maternal megadose of vitamin A.

# Theorized Mechanisms of Action

## **Table S21: Theorized mechanisms of action for the nonspecific BCG and DTP effects and interactions**

| Exposure | Theorized Mechanism |
| --- | --- |
| BCG | A meta-analysis of 13 studies in humans found a trend of increased IFN-$\gamma$ levels associated with BCG vaccinated individuals compared to non-vaccinated individuals, which may be indicative of a larger Th1 response and thus improved antibacterial and antiviral immune function after vaccination.(6) |
| DTP | Some biologic evidence suggests that this may be due to the pertussis toxin, which can change the Th1/Th2 cytokine profile resulting in depressed immune response to pathogens.(7, 8) Another possible mechanism is that DTP vaccination modifies signaling of toll-like receptor 4, associated with the innate immune system.(9) |
| DTP modified by infant sex | There is biological evidence that sex is associated with differences in antibody response to vaccines in infancy.(10) A possible mechanism is that DTP modifies the Th1/Th2 profile differently by sex.(9) |
| DTP modified by  vitamin A supplementation | Theoretically, this interaction may be due to vitamin A amplifying the immune effect of the vaccinations. A secondary analysis of data from a clinical trial in Guinea Bissau suggested that vitamin A supplementation was associated with lower TNF and interleukin (IL)-10 levels in male infants without DTP vaccination and higher TNF and interleukin (IL)-10 levels in female infants with DTP vaccination, with a significant 3-way interaction between sex, vitamin A supplementation, and DTP vaccination on TNF and interleukin levels.(11) |

**References:**

1. Jensen H, Benn CS, Lisse IM, Rodrigues A, Andersen PK, Aaby P. Survival bias in observational studies of the impact of routine immunizations on childhood survival. Trop Med Int Health. 2007;12(1):5-14.

2. Rothman KJ, Greenland S, Lash TL. Modern epidemiology. 3rd ed. Philadelphia: Wolters Kluwer Health/Lippincott Williams & Wilkins; 2008. x, 758 p. p.

3. Zhou Z, Rahme E, Abrahamowicz M, Pilote L. Survival bias associated with time-to-treatment initiation in drug effectiveness evaluation: a comparison of methods. Am J Epidemiol. 2005;162(10):1016-23.

4. Giobbie-Hurder A, Gelber RD, Regan MM. Challenges of guarantee-time bias. J Clin Oncol. 2013;31(23):2963-9.

5. Hernan MA, Sauer BC, Hernandez-Diaz S, Platt R, Shrier I. Specifying a target trial prevents immortal time bias and other self-inflicted injuries in observational analyses. J Clin Epidemiol. 2016;79:70-5.

6. Kandasamy R, Voysey M, McQuaid F, de Nie K, Ryan R, Orr O, et al. Non-specific immunological effects of selected routine childhood immunisations: systematic review. BMJ. 2016;355:i5225.

7. Lindblad EB. Aluminium compounds for use in vaccines. Immunol Cell Biol. 2004;82(5):497-505.

8. Ryan M, McCarthy L, Rappuoli R, Mahon BP, Mills KH. Pertussis toxin potentiates Th1 and Th2 responses to co-injected antigen: adjuvant action is associated with enhanced regulatory cytokine production and expression of the co-stimulatory molecules B7-1, B7-2 and CD28. Int Immunol. 1998;10(5):651-62.

9. Noho-Konteh F, Adetifa JU, Cox M, Hossin S, Reynolds J, Le MT, et al. Sex-Differential Non-Vaccine-Specific Immunological Effects of Diphtheria-Tetanus-Pertussis and Measles Vaccination. Clin Infect Dis. 2016;63(9):1213-26.

10. Zimmermann P, Perrett KP, Ritz N, Flanagan KL, Robins-Browne R, van der Klis FRM, et al. Biological sex influences antibody responses to routine vaccinations in the first year of life. Acta Paediatr. 2020;109(1):147-57.

11. Jorgensen MJ, Fisker AB, Sartono E, Andersen A, Erikstrup C, Lisse IM, et al. The effect of at-birth vitamin A supplementation on differential leucocyte counts and in vitro cytokine production: an immunological study nested within a randomised trial in Guinea-Bissau. Br J Nutr. 2013;109(3):467-77.
